# Pan-cancer analyses confirmed the cuproptosis-related gene FDX1 as an immunotherapy predictor and prognostic biomarker

**DOI:** 10.3389/fgene.2022.923737

**Published:** 2022-08-05

**Authors:** Chi Zhang, Yuanxiao Zeng, Xiuchen Guo, Hangjing Shen, Jianhao Zhang, Kaikai Wang, Mengmeng Ji, Shengwei Huang

**Affiliations:** ^1^ The First Clinical Medical College, Wenzhou Medical University, Wenzhou, China; ^2^ Operating Room, The First Affiliated Hospital of Wenzhou Medical University, Wenzhou, China; ^3^ Zhejiang Provincial Key Laboratory of Aging and Neurological Disorder Research, Department of Neurosurgery, The First Affiliated Hospital of Wenzhou Medical University, Wenzhou, China

**Keywords:** pan-cancer, cuproptosis, Fdx1, immunotherapy, drug resistance

## Abstract

**Background:** The latest research identified cuproptosis as an entirely new mechanism of cell death. However, as a key regulator in copper-induced cell death, the prognostic and immunotherapeutic value of FDX1 in pan-cancer remains unclear.

**Methods:** Data from the UCSC Xena, GEPIA, and CPTAC were analyzed to conduct an inquiry into the overall differential expression of FDX1 across multiple cancer types. The expression of FDX1 in GBM, LUAD and HCC cell lines as well as their control cell lines was verified by RT-QPCR. The survival prognosis, clinical features, and genetic changes of FDX1 were also evaluated. Finally, the relationship between FDX1 and immunotherapy response was further explored through Gene Set Enrichment Analysis enrichment analysis, tumor microenvironment, immune cell infiltration, immune gene co-expression and drug sensitivity analysis.

**Results:** The transcription and protein expression of FDX1 were significantly reduced in most cancer types and had prognostic value for the survival of certain cancer patients such as ACC, KIRC, HNSC, THCA and LGG. In some cancer types, FDX1 expression was also markedly correlated with the clinical characteristics, TMB, MSI, and antitumor drug susceptibility or resistance of different tumors. Gene set enrichment analysis showed that FDX1 was significantly associated with immune-related pathways. Moreover, the expression level of FDX1 was confirmed to be strongly correlated with immune cell infiltration, immune checkpoint genes, and immune regulatory genes to a certain extent.

**Conclusion:** This study comprehensively explored the potential value of FDX1 as a prognostic and immunotherapeutic marker for pan-cancer, providing new direction and evidence for cancer therapy.

## 1 Introduction

As a major public health problem worldwide, cancer is a significant barrier to increasing life expectancy with its fast-growing incidence and mortality (Bray et al., 2021; Sung et al., 2021). The situation of cancer treatment continues to be extremely serious. Tumors are characterized by complex biological processes, such as proliferative signaling, evading growth suppressors, resisting cell death, enabling replication immortality, inducing angiogenesis, and activating invasion and metastasis (Hanahan and Weinberg, 2011). Furthermore, there is an ongoing interaction between tumor invasion and the host immune response, which was strongly correlated with tumor progression. The immune system is essential in control of tumors, and effective immunotherapy could be achieved through preexisting adaptive immune responses within tumors, such as checkpoint inhibitors (Bruni et al., 2020a). In view of the prevalence of tumors and the convoluted process of tumorigenesis, it is of great significance to explore in-depth the expression of relevant genes in pan-cancer and to assess their levels for clinical treatment and prognostic prediction (Qin et al., 2022).

Recently, a previously uncharacterized cell death mechanism, named as copper-induced death (cuproptosis), was newly unmasked (Tsvetkov et al., 2022). This study demonstrated that copper could bind to and aggregate with lipoylated TCA cyclins, which then trigger proteotoxic stress along with the loss of Fe-S cluster proteins, resulting in cell death (Li et al., 2022). In fact, in the past 10 years of research, there was no shortage of studies on the mechanism of copper-induced cell death. For example, copper ions can induce autophagy levels in testicular cells via the AMPK-mTOR pathway (Guo et al., 2022a). In addition, studies have found that the immunotoxicity caused by exposure to excessive copper could cause apoptosis in multiple organs throughout the body, such as spleen (Guo et al., 2022b), thymus (Mitra et al., 2012; Mitra et al., 2013), liver (Keswani et al., 2015), lung (Jian et al., 2020) and other tissues. In the latest related research, as an upstream regulator of protein lipoylation, FDX1 had been confirmed to be the key regulator of copper-induced death (Tsvetkov et al., 2022). As is known, FDX1 is one of the carriers of ferricoxigenin reductase (FDXR) (Hanukoglu and Jefcoate, 1980), with the other carrier being FDX2. Previous studies showed that the actions of FDX1 and FDX2 were relatively highly specific. Among them, FDX1, which is the core of our study in this article, catalyzes more predominantly the core reaction of steroidogenesis (Sheftel et al., 2010; Shi et al., 2012; Cai et al., 2017; Peng et al., 2017), whilst also providing electrons to cytochrome P450 enzymes as part of vitamin D metabolism (Ewen et al., 2012). In the past decade, the link between FDXR and cancer had likewise been explored (Yu et al., 2003; Ichikawa et al., 2006; Zhang et al., 2020). However, as for FDX1, its role in cancer was poorly understood, and it had only been proven to affect the prognosis of lung adenocarcinoma and mediate its metabolism (Zhang et al., 2021).

This new mechanism shed new light on us. In this study, we performed a comprehensive analysis of FDX1 expression in pan-cancer, including differentially expressed gene (DEG) analysis, protein expression analysis, prognostic analysis and enrichment analysis of different tumor types, etc. Finally, the correlation of FDX1 expression with immune infiltrating cells, immune regulators and drug sensitivity was compared. The results not only suggested that FDX1 might be a potent prognostic biomarker, which was closely associated with cancer immunomodulatory mechanisms and resistance to antitumor drugs, but also revealed its as a potential predictor of pan-cancer immunotherapy.

## 2 Result

### 2.1 Analysis of FDX1 expression in pan-cancers

**FIGURE 1:**
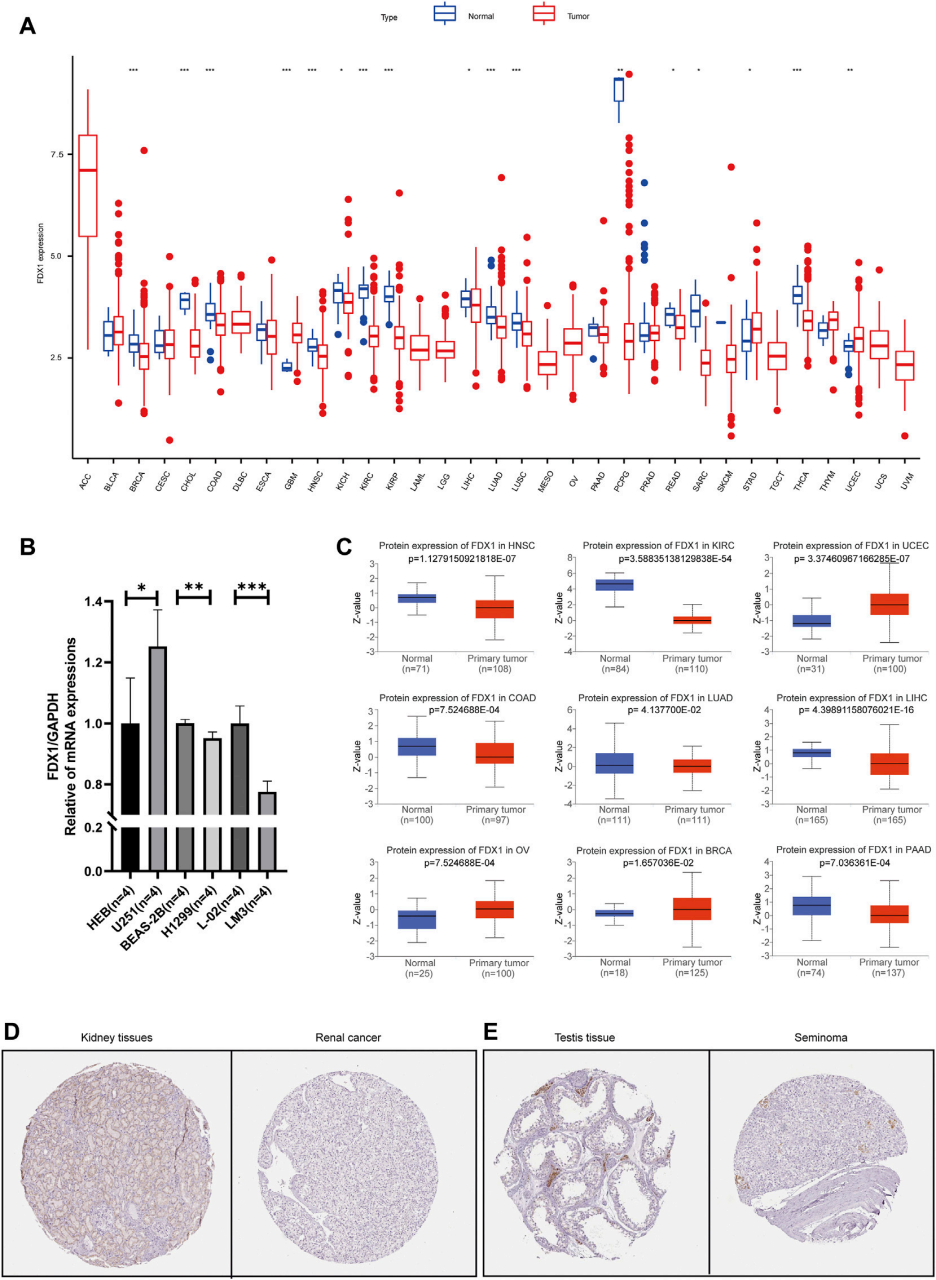
Differential expression of FDX1 between normal and tumor tissue samples **(A)** FDX1 expression in pan-cancers and their corresponding normal samples from USCS Xena **(B)** The mRNA level of FDX1 was highly expressed in U251 (GBM) cell line compared to HEB cell line (normal). However, the expressions in BEAS-2B and L-02 cell lines (normal) were higher than those in H1299 (LUAD) and LM3 (HCC) cell lines, respectively **(C)** The differential protein levels of FDX1 in different tumors. Validation of immunohistochemical picture results at the protein level of FDX1 **(D)** Normal kidney and renal cancer **(E)** Normal testis and seminoma. The red boxplot represents tumor (T) and the blue boxplot represents normal tissue (N).

The differential analysis of FDX1 based on cancer and paracancerous tissue samples from the TCGA database indicated that FDX1 had evidently higher expression in paraneoplastic tissues than cancer tissues, such as BRCA, CHOL, COAD, HNSC, KICH, KIRC, KIRP, LIHC, LUAD, LUSC, PCPG, READ, SARC and THCA (Figure 1A). For cancers lacking paracancerous tissue, we found no significant difference except DLBC (Supplementary Figure S1). The expression of FDX1 was further verified by qRT-PCR in GBM, LUAD and HCC cell lines (Figure 1B). The results showed that the FDX1 mRNA level in GBM cell line (U251) was significantly higher than that in normal human astrocyte cell line (HEB). The expression of FDX1 in LUAD (H1299) and HCC (LM3) cell lines was lower than that in human bronchial epithelial cells (BEAS-2B) and normal human liver cell line (L-02). The results of qRT-PCR experiments were consistent with bioinformatics. FDX1 expression level in pan-cancers indicated the probable links between FDX1 and cancers.

The protein levels of FDX1 in different cancers showed that expression of FDX1 decreased in solid tumors such as HNSC, KIRC, COAD, LUAD, LIHC, PAAD. However, it was very interesting that FDX1 were significantly higher in FDX1 in UCEC, OV, BRCA and other female reproductive system cancers (Figure 1C). Moreover, we found the level of FDX1 in renal carcinoma and seminoma was lower than that in normal tissues (Figure 1D).

### 2.2 Prognostic value of FDX1 in cancer patients

**FIGURE 2:**
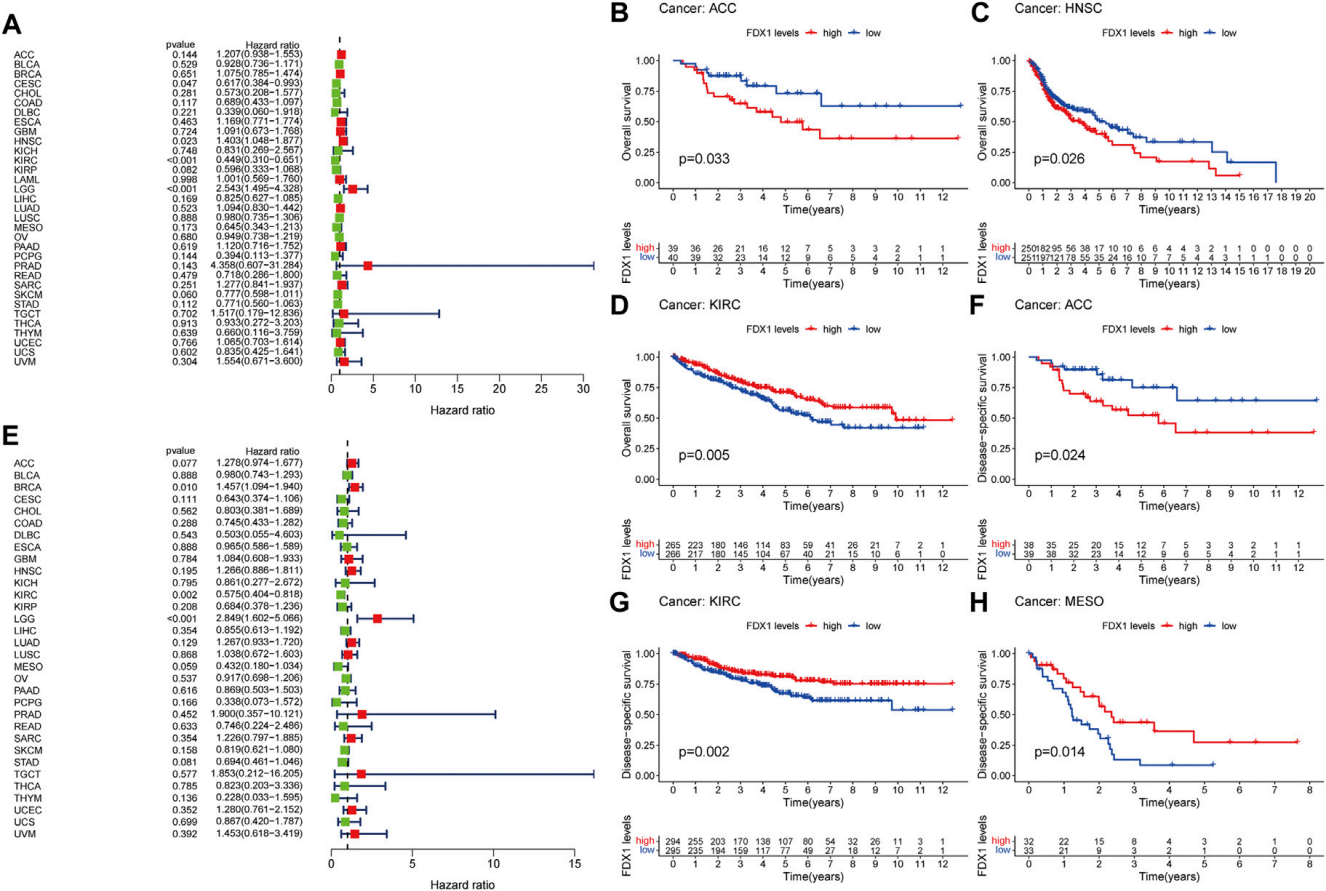
Prognostic assessment of FDX1 expression in OS and DFS. Correlation between FDX1 expression and OS **(A)** and DFS **(E)** by utilizing Cox proportional hazards model **(B–D)** Kaplan-Meier analysis of OS in patients with high and low FDX1 expression **(F–H)** Kaplan-Meier analysis of DFS in patients with high and low FDX1 expression. HR (hazard ratio) > 1 indicates that FDX1 may be an adverse factor in the occurrence and development of cancer, and it is shown in red; 0 < HR < 1 indicates that FDX1 may be a protective factor in cancer, and it is shown in green.

The potential prognostic value of FDX1 was assessed using Cox proportional hazards model and Kaplan Meier analysis. The results of Cox model showed that the expression level of FDX1 was negatively associated with prognosis of CESC (*p* = 0.047) and KIRC (*p* < 0.001), as well as positively in HNSC (*p* = 0.023) and LGG (*p* < 0.001) (Figure 2A). Kaplan-Meier analysis showed that high expression of FDX1 predicted poor OS in ACC (Figure 2B, *p* = 0.033), HNSC (Figure 2C, *p* = 0.026), PAAD (Supplementary Figure S2B, *p* = 0.038) and LGG (Supplementary Figure S2C, *p* < 0.001), while high FDX1 predicted better OS in KIRC (Figure 2D, *p* = 0.005), COAD (Supplementary Figure S2A, *p* = 0.019) and SKCM (Supplementary Figure S2D, *p* = 0.040).

For DSS, high expression of FDX1 was a negative factor in BRCA (*p* = 0.010) and LGG (*p* < 0.001) patients, but a positive factor in KIRC patients (*p* = 0.002; Figure 2E). Consistent with the results of the Cox proportional hazards model of DSS, the K-M curve indicated that high level of FDX1 was positively correlated with good survival outcomes in KIRC (Figure 2G, *p* = 0.002) and MESO (Figure 2H, *p* = 0.014), and negatively correlated with survival in ACC (Figure 2F, *p* = 0.024) and LGG (Supplementary Figure S2E, *p* < 0.001).

**FIGURE 3:**
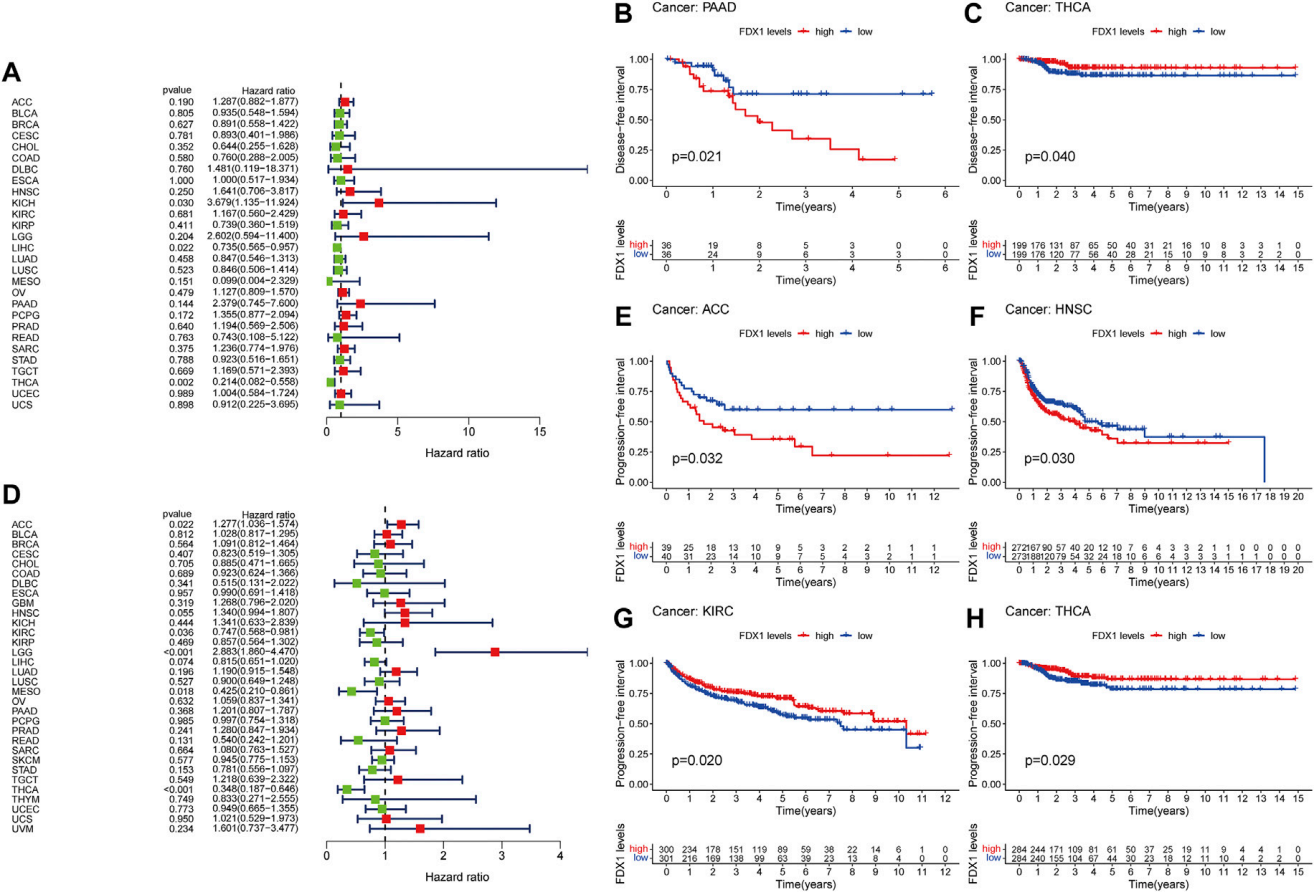
Prognostic assessment of FDX1 expression in DFI and PFI. Correlation between FDX1 expression and DFI **(A)** and PFI **(D)** by utilizing Cox proportional hazards model **(B,C)** Kaplan-Meier analysis of DFI in patients with high and low FDX1 expression **(E–H)** Kaplan-Meier analysis of PFI in patients with high and low FDX1 expression. HR (hazard ratio) > 1 indicates that FDX1 may be an adverse factor in the occurrence and development of cancer, and it is shown in red; 0 < HR < 1 indicates that FDX1 may be a protective factor in cancer, and it is shown in green.

Forest plot showed that high expression of FDX1 predicted poor DFI in KICH (*p* = 0.030) and better DFI in LIHC (Figure 3A, *p* = 0.022). However, Kaplan-Meier analysis found that KICH is not statistically significant. In addition, the K-M curve of PAAD (Figure 3B, *p* = 0.021) showed that high expression of FDX1 indicated poor prognosis. While in early THCA (Figure 2C, *p* = 0.040) and LIHC (Supplementary Figure S1F, *p* = 0.029), high expression of FDX1 indicated good prognosis.

Furthermore, in the PFI-related Cox proportional hazards model, FDX1 also exhibited significantly prognostic value in ACC (*p* = 0.022), KIRC (*p* = 0.036), LGG (*p* < 0.001), MESO (*p* = 0.018), THCA (*p* < 0.001; Figure 3D). Patients with high expression of FDX1 had prolonged PFI in KIRC (Figure 3G, *p* = 0.020), THCA (Figure 3H, *p* = 0.029), and LIHC (Supplementary Figure S2H, *p* = 0.035), but shortened in ACC (Figure 3E, *p* = 0.032), HNSC (Figure 3F, *p* = 0.030), and LGG (Supplementary Figure S2H, *p* < 0.001).

### 2.3 The relationship between FDX1 and clinical information

**FIGURE 4:**
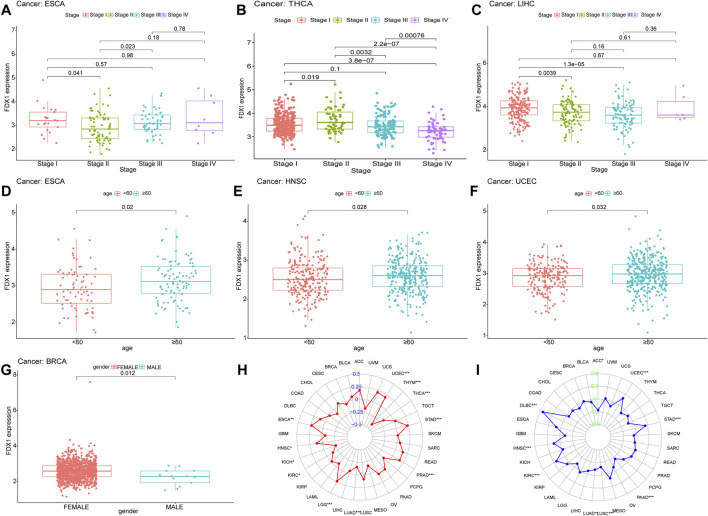
Analysis of clinic correlation of FDX1 expression. Correlation analysis of FDX1 expression and tumor clinical stage **(A)**ESCA **(B)**THCA **(C)**LIHC. The red, green, blue, and purple boxplots represent stage I-IV, respectively. Correlation analysis of FDX1 expression and age (with 60 years as the cutoff) of cancer patients **(D)**ESCA **(E)**HNSC **(F)**UCEC. Red means the patient is younger than 60 years old, and blue means the patient is 60 years or older **(G)** Correlation analysis of FDX1 expression with gender in cancer patients. Relationship of FDX1 expression with TMB **(H)** and MSI **(I)**.

In the advanced stages of THCA, especially in stage III and stage IV, the expression of FDX1 was significantly lower than early stages (Figure 4B). In ESCA, the expression of FDX1 was the lowest in stage II, which was markedly different from stages I and III. It might be linked to the excessive proliferation and invasion of stage II cancer cells and cell death inhibition (Figure 4A) (Weaver et al., 2014). In the stage II and stage III of LIHC, FDX1 was lower compared to the stage I. However, sample size of stage IV LIHC was too small to indicated the comparative results, effectively (Figure 4C). Furthermore, the expression of FDX1 was higher in HNSC, UCEC and ESCA patients under 60 (Figures 4D–F). And FDX1 was lower in male patients in BRCA (Figure 4G).

### 2.4 Relationship of FDX1 with TMB and MSI

More and more studies have suggested that TMB and MSI could be independent biomarkers reflecting the efficacy of immune checkpoint inhibitor therapy (Rizzo et al., 2021) and the prognosis of cancers (Condelli et al., 2021). Thence, we further explored the relationship of FDX1 with TMB and MSI in the pan-cancer cohort and the detailed results were presented in Supplementary Table S1 and Supplementary Table S2. FDX1 expression was positively related to the TMB in UCEC, STAD, PRAD, LGG, HNSC and ESCA, whereas the negative association was observed in THYM, THCA, LUAD, KIRC and KICH (Figure 4H). For MSI, the positive association was obtained in DLBC, UCEC, STAD, KIRC and HNSC, as well as the negative association was in ACC, PAAD, LUSC and LUAD was identified (Figure 4I).

### 2.5 GSEA of FDX1 in HALLMARK pathways

**FIGURE 5:**
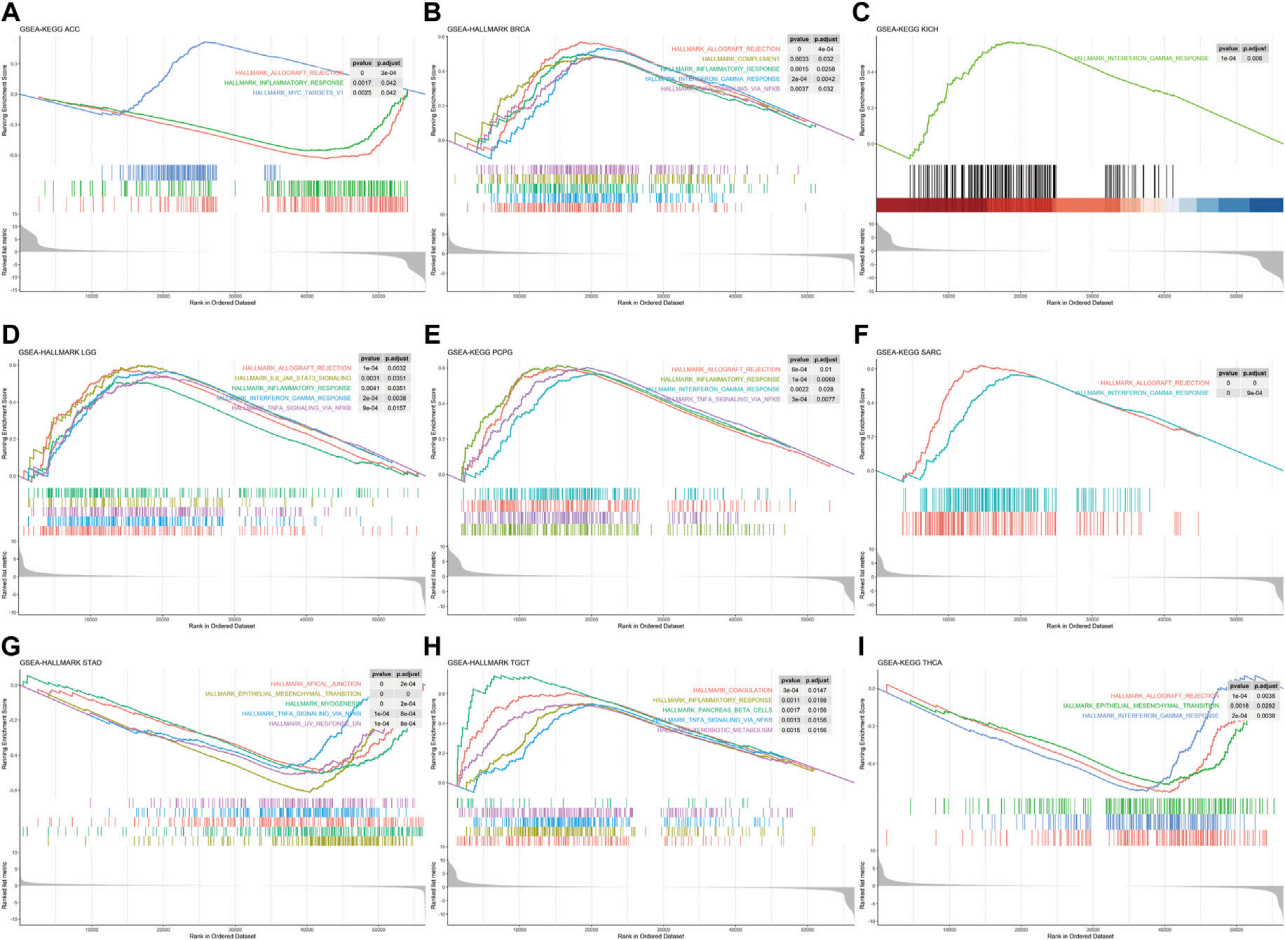
Gene set enrichment analysis of FDX1 in Pan-cancer. HALLMARK enrichment results associated with FDX1 expression **(A)**ACC **(B)**BRCA **(C)**KICH **(D)**LGG **(E)**PCPG **(F)**SARC **(G)**STAD **(H)**TGCT **(I)**THCA. The *X*-axis represents the sequencing of expression values of genes enriched in different pathways within the sample, and the *Y*-axis represents the enrichment score.

Single-gene GSEA was used to identify relevant pathways affected by FDX1 expression in pan-cancer. The results showed that FDX1 was positively related with immune-related pathways in BRCA, KICH, LGG, PCPG, SARC, and TCGT such as inflammatory response, interferon-gamma response, TNF-A signaling *via* NFKB and allograft rejection. Conversely, the aforementioned pathways in ACC, STAD and THCA were negatively regulated (Figure 5). Moreover, FDX1 was positively enriched in oxidative phosphorylation and/or fatty acid metabolism pathways in ESCA, KIRC, KIRP, LIHC, LUSC, PRAD and THYM (Supplementary Figure S3). Furthermore, we observed that the epithelial-mesenchymal transition pathway exhibited negative enrichment in STAD, THCA, BLCA, COAD and LUAD (Supplementary Figure S4). Our findings suggested that FDX1 generally correlated with many important pathways in cancer formation.

### 2.6 Correlation between FDX1 expression and immune infiltrating level in pan-cancers

**FIGURE 6:**
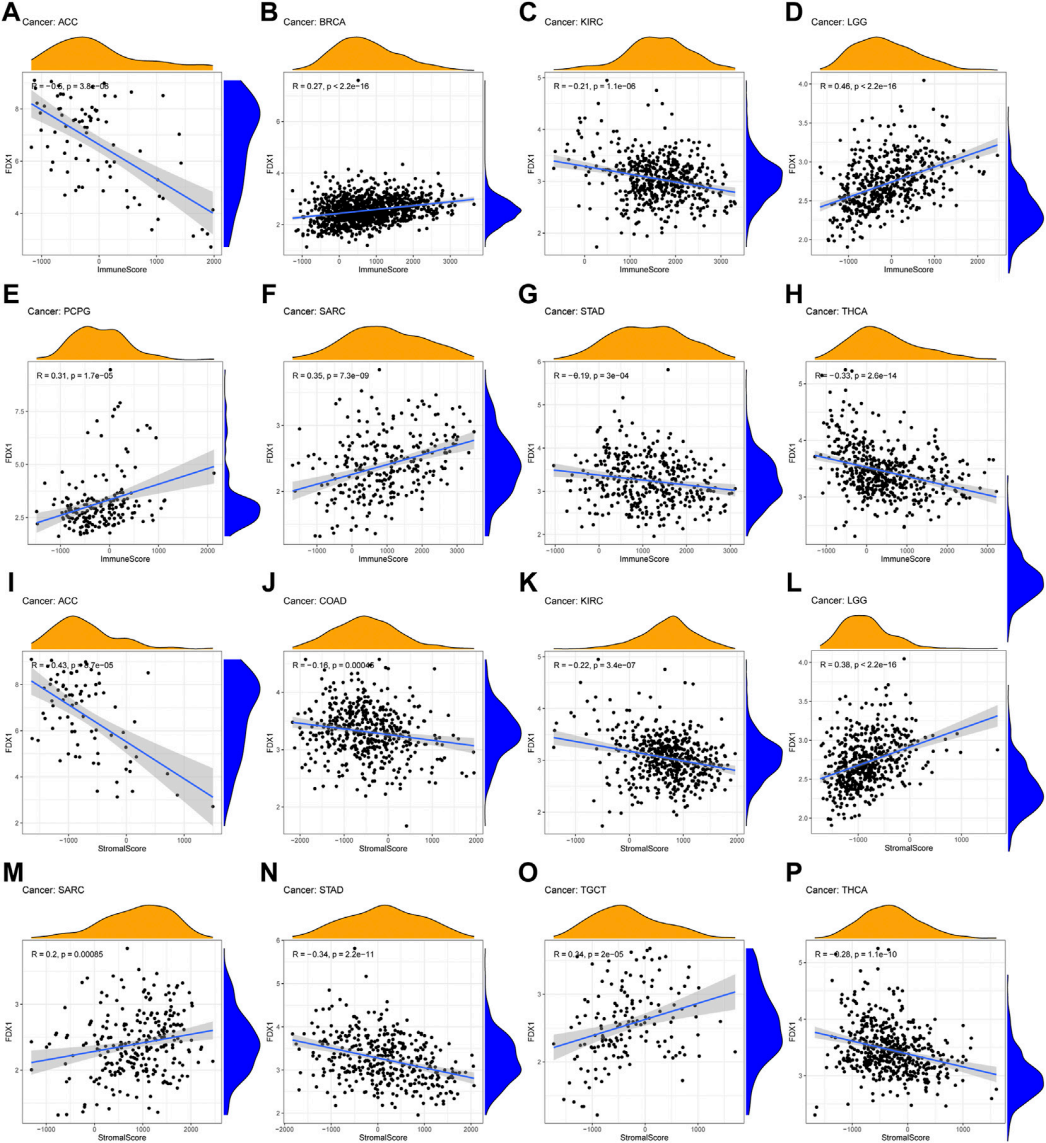
Association of FDX1 with the TME Composition **(A–H)** Immune score and **(I–P)** stromal score were analyzed to reveal the immune and stromal composition in TME.

According to GSEA, we observed the underlying association between FDX1 and immune-related factors. Therefore, tumor microenvironment and immune infiltrate analysis were performed. The results showed that FDX1 had positive correlation with the immune score in BRCA (R = 0.27), LGG (R = 0.46), PCPG (R = 0.31) and SARC (R = 0.35) (Figures 6D–F). For stromal scores, positive correction with FDX1 was identified in LGG (R = 0.38), SARC (R = 0.2) and TGCT (R = 0.34) (Figure 6L, 6M, 6O). FDX1 was negatively correlated with the stromal score of COAD (R = −0.16, Figure 6J). In ACC (immune scores: R = −0.6, stromal score: R = −0.43), KIRC (immune scores: R = −0.21, stromal score: R = −0.22), STAD (immune scores: R = −0.19, stromal score: R = −0.34) and THCA (immune scores: R = −0.33, stromal score: R = −0.28), FDX1 transcript levels were consistently negatively correlated with immune and stromal scores (Figure 6).

**FIGURE 7:**
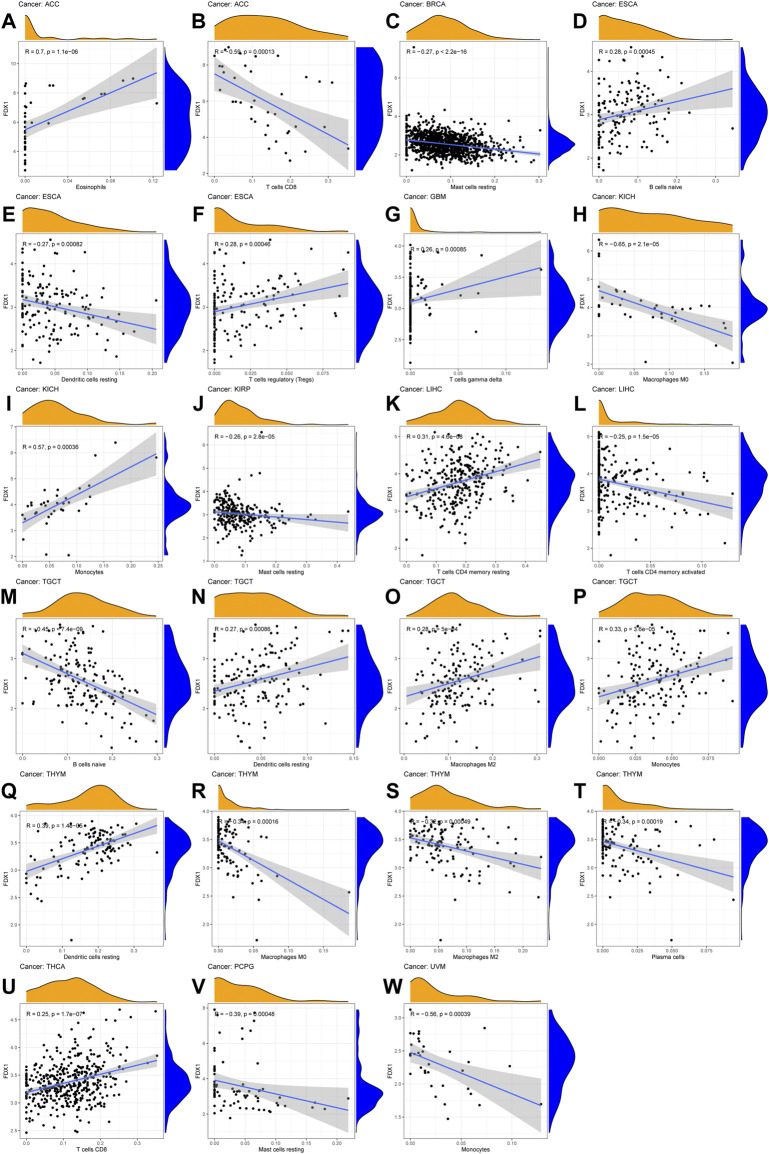
The correlation between FDX1 expression and immune cell infiltration **(A–W)** Scatter plots showing that tumor-infiltrating immune cells were significantly correlated with FDX1 expression.

Immune-related cell infiltration is the main mechanism affecting the tumor microenvironment, so we further investigated the relationship between FDX1 expression and immune infiltration analysis in pan-cancer. We found that FDX1 was associated with infiltration levels of T cells in nine cancer types, dendritic cells in seven cancer types, monocytes-macrophages in seven cancer types and mast cells in five cancer types. In particular, the expression level of FDX1 was well correlated with six types immune-related cellular infiltration (including CD8^+^ T cells, CD4^+^ T cells, dendritic cells, mast cells) in BRCA, six types (including T cells, monocytes, macrophages, mast cells) in renal cancer, five types (including CD8^+^ T cells, regulatory T cells, dendritic cells, mast cells) in THCA, and four types (including plasma cells, macrophages, dendritic cells) in TGCT. The results of immune cell infiltration with |R|>0.25 were shown in Figure 7, and the rest were shown in Supplementary Figure S5.

### 2.7 FDX1 correlated with the majority of cuproptosis-related genes, immuneregulatory genes and chemokines

**FIGURE 8:**
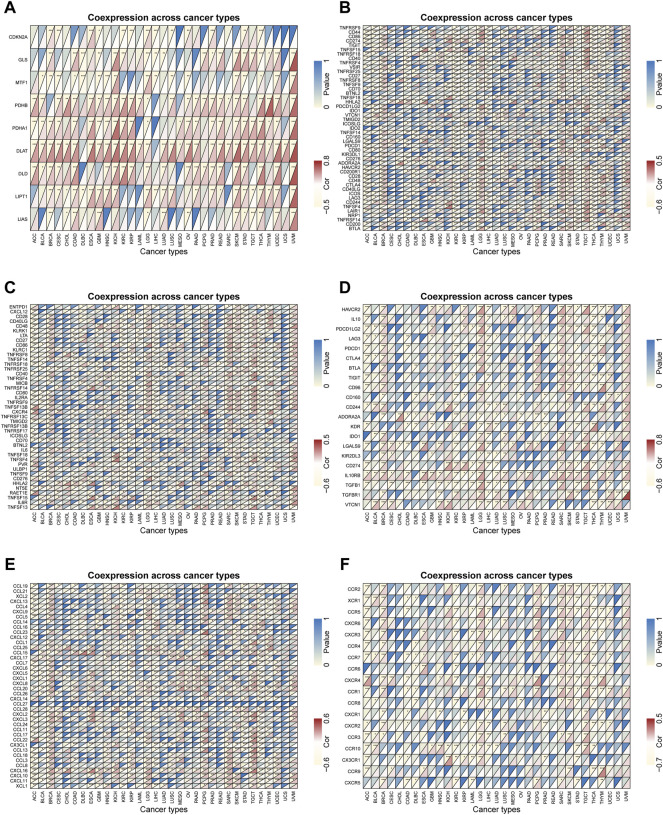
Gene co-expression analysis of FDX1 in pan-cancer. The heatmaps presenting the correlations of FDX1 expression with genes related to **(A)** cuproptosis **(B)** immune checkpoint **(C)** immune-activation **(D)** immunosuppressive status **(E)** chemokines proteins and **(F)** chemokine receptors. The upper left corner of each square represents the *p* value, * represents *p* < 0.05, ** represent *p* < 0.01, and *** represent *p* < 0.001; the lower right corner represents the correlation between FDX1 and other genes, red represents positive correlation, and yellow represents negative correlation.

In view of the above results, FDX1 might have a non-negligible relationship with tumor immune regulation, so we further explored the role of FDX1 at the gene level. We performed comprehensive co-expression analysis on the cuproptosis-related genes, immune checkpoint genes, immune-activating genes, immunosuppressive status-related genes, chemokines and chemokine receptors. The results showed that cuproptosis-related genes such as PDHB, PDHA1, DLAT, DLD, LIPT1 were significantly positively correlated with FDX1 in pan-cancer (Figure 8A). For immune-regulatory genes and chemokines, the gene co-expression of FDX1 showed significant heterogeneity in different types of cancer, but there was an interesting consistency in the immune gene level. For instance, the results of 47 immune checkpoint genes showed that most genes in BRCA, LGG, SARC, SKCM, TGCT and UVM were significantly positively correlated with FDX1, while negatively correlated in ACC, KIRC, LIHC, STAD and THCA (Figure 8B). In addition, the correlation analysis of immune-activating genes, immunosuppressive status-related genes, chemokines and chemokine receptors found that the results were unexpectedly consistent with the previous ones (Figures 8C–F). Therefore, FDX1 might play a crucial role in the immune regulation of tumors. What’s more, in view of the previous GSEA results, we also performed a correlation analysis of the cellular redox signaling-related genes with a score greater than 25 in the genecards. It was found that FDX1 was also well correlated with many cellular redox signaling-related genes in pan-cancer, for example, most genes in LGG and DLBC were significantly negatively correlated with FDX1 but positively correlated in UVM and SKCM (Supplementary Figure S6).

### 2.8 Drug resistance analysis and molecular docking

**FIGURE 9:**
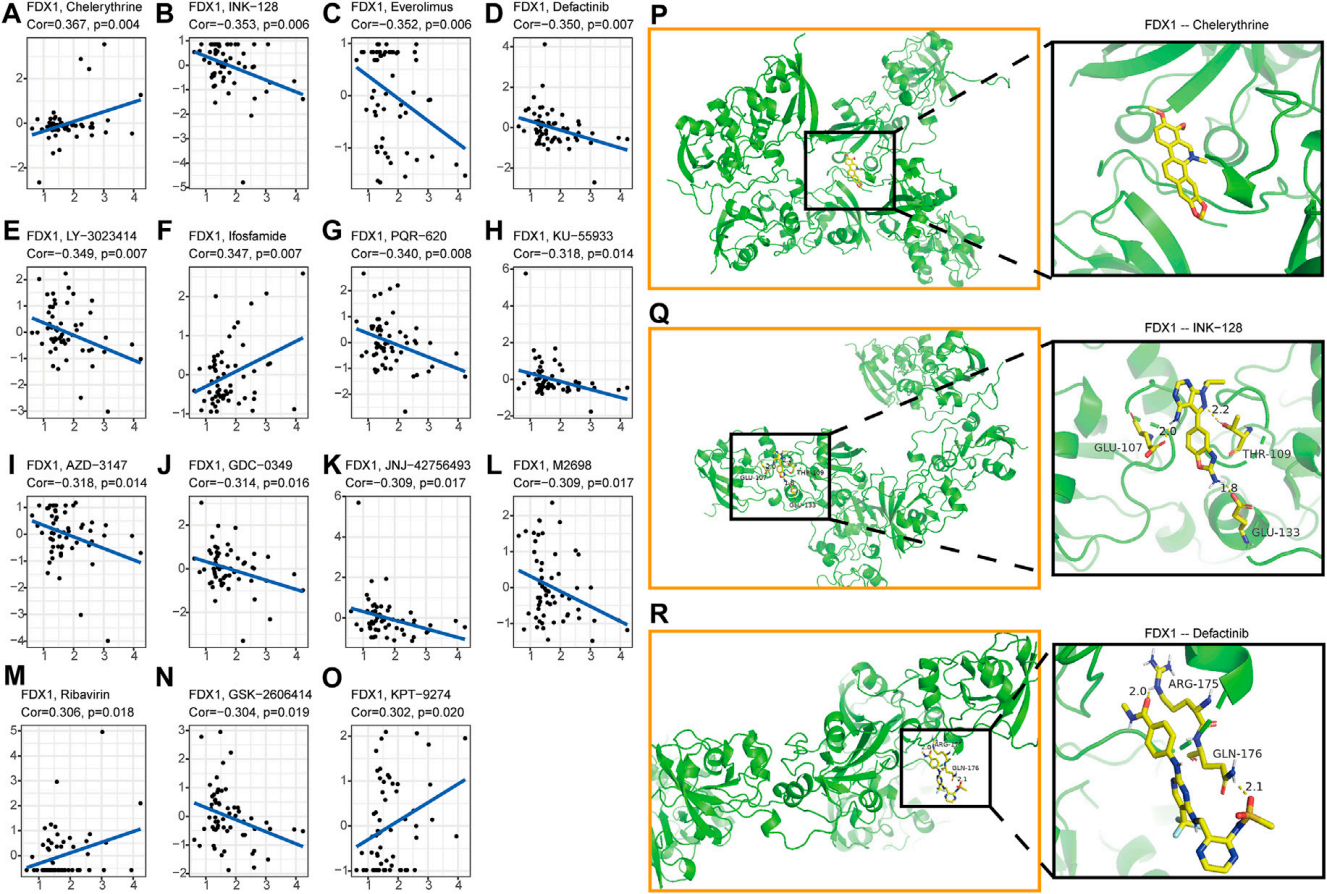
Analysis of FDX1 Expression and Sensitivity of Tumor Chemotherapeutics **(A)**Chelerythrine **(B)**INK-128 **(C)**Everolimus **(D)**Defactinib **(E)**LY-3023414 **(F)**Ifosfamide **(G)**PQR-620 **(H)**KU-55933 **(I)**AZD-3147 **(J)**GDC-0349 **(K)**JNJ-42756,493 **(L)**M2698 **(M)**Ribavirin **(N)**GSK-2606414 **(O)**KPT-9274. The *X*-axis represents the gene expression level of FDX1, and the *Y*-axis represents the z scores of Compound activity. Molecular docking results of protein FDX1 (3P1M) with Chelerythrine **(P)**, INK-128 **(Q)** and Defactinib **(R)**. No hydrogen bond was found between FDX1 and Chelerythrine, but there was a stable binding energy. There are three hydrogen bonds of 2.0, 2.2 and 1.8 Å in the docking between FDX1 and INK-128. The docking results of FDX1 and Defactinib showed two hydrogen bonds of 2.0 and 2.1 Å respectively.

Nowadays, the tumor drug resistance has been paid more and more attention. Finally, we investigated the potential correlation analysis between pan-cancer drug resistance and FDX1 expression. Fifteen drugs with |R|>0.3 in 59 tumor cell lines were identified (Supplementary Table S3). Among them, FDX1 expression level was positively correlated with the sensitivity of Chelerythrine, Ifosfamide, Ribavirin, and KPT-9274 (Figures 9A,F, M, O). While FDX1 expression level was negatively correlated with multiple drug sensitivities including GSK-2606414, M2698, JNJ-42756,493, GDC-0349, AZD-3147, KU-55933, PQR-620, LY-3023414, Defactinib, Everolimus, and INK-128 (Figures 9B–E,G–K, L, N). In view of the good correlation between chemotherapeutic drug sensitivity and FDX1, we used the molecular docking method to further determine the potential targeting effect of chemotherapeutic drugs on FDX1. Among them, the binding free energy of FDX1 and Chelerythrine is -7.04 kcal/mol (Figure 9P), and there are three hydrogen bonding forces between INK-128 and FDX1 (Figure 9Q), and two hydrogen bonding forces between Defactinib and FDX1(Figure 9R). The results showed that a variety of chemotherapeutic drugs exhibited excellent binding activity to FDX1.

## 3 Discussion

This study revealed the survival predictive value and potential immunotherapy value of FDX1 in pan-cancer with comprehensive analyses. Our study found that the gene and protein levels of FDX1 were significantly lower in most tumors and had certain prognostic value in some cancers. In addition, the expression level of FDX1 was closely associated with immune and inflammation-related pathways, immune cell infiltration, and various immune-related genes. FDX1 was also associated with sensitivity to cancer chemotherapy drugs. Consequently, FDX1 might be a potential prognostic biomarker and predictor for immunotherapy.

Cuproptosis is a pattern of cell death newly defined by GOLUB *et al.* (Tsvetkov et al., 2022), and its mechanism is distinct from other mechanisms known to regulate cell death. Some previous studies had demonstrated that unbalanced copper homeostasis could induce cell death and exert anti-tumor effects (Jiang et al., 2022), for instance, disulfiram/copper complexes can induce apoptosis in non-small cell lung cancer cells (Wu et al., 2018), induce autophagic death in colorectal cancer cells (Hu et al., 2021) and induce ROS-dependent apoptosis in malignant prostate cells (Safi et al., 2014). However, the current research on cuproptosis is still in its infancy. Its clear system and its specific regulatory mechanism for various cancers remains to be further explored. Among them, the loss of FDX1, as a key gene in copper ionophores-induced death, makes cells resistant to copper-induced cell death (Tsvetkov et al., 2022), which is consistent with our finding that FDX1 is under expressed in many cancers. The detailed functions of FDX1 have not been elucidated in many cancers, and only Zhang *et al.* (Zhang et al., 2021) reported that FDX1 deficiency leaded to numerous metabolic changes in LUAD, as well as changed in tumor-associated inflammation and immune microenvironment, which also corroborated with our partial results. Based on the above evidence, we focused on the relevant mechanisms of FDX1 in wide varieties of tumors, and conducted in-depth exploration at the pan-cancer level.

Combining the assessment of FDX1 mRNA levels in 33 human tumors from both databases, we observed that its expression was significantly declined in majority of tumors. In general, the protein expression level can better reflect the activity and function of FDX1. Unfortunately, due to the lack of relevant data in public databases, only partial tumors were analyzed for protein levels. By comparing transcription levels with protein levels, it was found that transcription levels matched overall activity in HNSC, KIRC, UCEC, COAD, LUAD, LIHC and PAAD. However, in some cancers, such as BRCA and OV, the two were not consistent, which might be related to post-transcriptional protein modification or metabolism. The expression of FDX1 was not significantly different in most cancers by clinical stage, age, and gender, but in some cancers, especially ACC, KIRC, HNSC, MESO, and THCA, it could serve as an independent prognostic factor and a potential prognostic marker.

Furthermore, according to the GSEA enrichment results, we found FDX1 was closely related to immune and inflammatory responses, oxidative phosphorylation, fatty acid metabolism and endothelial-mesenchymal transition. This was consistent with existing tumor mechanisms (Li et al., 2011; Currie et al., 2013; Gonzalez and Medici, 2014; Ashton et al., 2018). Among them, we paid special attention to multiple immune pathways, including the inflammatory response, interferon gamma response, TNF-a signaling pathway and IL-6/JAK/STAT3 signaling, all of which had been shown to be closely related to tumor development (Balkwill, 2009; Diakos et al., 2014; Alspach et al., 2019; Manore et al., 2022). To further investigate the potential value of FDX1, we explored the correlation of FDX1 with the tumor microenvironment and immune cell infiltration. The results showed that in the TME of many cancers, FDX1 had a good correlation with various types of immune cell invasion (CD4^+^ T cells, Macrophages, B cells, T cells regulatory, Mast cells resting, etc.). Therefore, we hypothesized that FDX1 may regulate immune cells within the tumor microenvironment through multiple pathways, rather than specifically targeting immune cells.

Under normal conditions, the immune system can utilize immune cell infiltration to recognize and eliminate tumor cells in the TME. The infiltration of immune cells and the induction of anti-tumor immune responses are mainly controlled by various chemokines, chemokine receptors, cytokines and immune checkpoints (Nagarsheth et al., 2017; Petitprez et al., 2020; Chen et al., 2021). Taking these into account, we evaluated the association of FDX1 with immune checkpoint genes, immune activation genes, immunosuppressive state-relative genes, chemokine genes, and chemokine receptor genes. Interestingly, FDX1 was significantly associated with most genes in ACC, BRCA, KIRC, LGG, LIHC, PCPG, SARC, SKCM, TGCT, THCA and other malignant tumors, and was highly consistent with the previous GSEA enrichment results. This suggested that FDX1 might serve as a potential regulatory target in immunotherapy for these cancers, and it also implied a non-negligible role in the induction of immune cell recruitment.

Tumor immune microenvironment plays an important role in the development, prognosis and immunotherapy of cancer (Hinshaw and Shevde, 2019; Lei et al., 2020). In ACC, FDX1 was significantly negatively correlated with immune score and immune cell infiltration. However, different immune cell types also have different effects on cancer, for example, CD8^+^ T cells are often associated with good prognosis while regulatory T cells are mostly associated with poor prognosis (Bruni et al., 2020b). Immune cell infiltration analysis found a significant negative correlation between FDX1 and CD8^+^ T cell infiltration, suggesting that ACC patients with high expression of FDX1 may have a worse prognosis, which was also mutually confirmed with our analysis results (Figures 2B,F, 3E). ACC is a rare but highly malignant tumor, and the treatment options for advanced cancer are severely limited (Fishbein et al., 2021). Therefore, immunotherapy such as checkpoint inhibitors and monoclonal antibodies may after all be accepted as an effective potential treatment for these patients (Jimenez et al., 2022). Interestingly, FDX1 was significantly negatively correlated with the expression of many immune checkpoint genes in ACC, especially PD-1 (PDCD1), PD-L1(CD274), CTLA4 and other important immunotherapy targets, which may also be closely related to the immune escape mechanism of ACC tumor cells (Jiang et al., 2019).

In addition, in terms of tumor chemotherapy, there were few studies on the relationship between FDX1 and tumor resistance. Only Tsvetkov *et al.* (Tsvetkov et al., 2019) found that FDX1 was highly associated with the proteasome inhibitor elesclomol and a direct target of elesclomol. However, no correlation was found between the two in our study, which might be related to the low number of cell lines in the CellMiner database and the slow update of experimental data. In any event, we found that FDX1 expression correlated with sensitivity to various drugs such as Chelerythrine, INK-128, Everolimus and Defactinib, and FDX1 might also play a role in chemotherapy. These speculations still require further experimental confirmation to test whether FDX1 may become a potential target and predictor of cancer immunotherapy.

In conclusion, we found FDX1 was a novel biomarker for diverse cancers. We found FDX1 had significant correlations with prognosis, mutation and immunity of pan-cancer. It expects to be a novel therapy target for multiple cancers.

## 4 Materials and methods

### 4.1 Data collection

All data were obtained from the UCSC Xena database (Goldman et al., 2020), where we downloaded gene expression data (FPKM was selected), mutation data, clinical data and overall survival data from the GDC hub, and acquired other survival data from the Pan-Cancer Atlas Hub.

### 4.2 FDX1 differential expression analysis

FDX1 mRNA differential expression levels of different cancer types were determined using R software (version:4.0.2), where the Wilcoxon test was used for testing and the “ggpubr” package was used to print box plots. Cancer and paracancer samples from the TCGA database were compared. For some cancers lacking paracancerous control samples, we integrated the TCGA data and the GTEx data from GEPIA website (Tang et al., 2017) (http://gepia.cancer-pku.cn/), and performed the differential expression analysis.

### 4.3 Cell culture

Human bronchial epithelial cells (BEAS-2B) and human LUAD cell lines (H1299) were purchased from the American Type Culture Collection (ATCC, United States). Normal human astrocyte cell line (HEB) was purchased from the Guangzhou Institute of Biomedicine and Health, Chinese Academy of Sciences. GBM cell lines (U251), normal human liver cell line (L-02) and HCC cell line (HCC-LM3) were received from Shanghai Institute of Cell Biology, Chinese Academy of Sciences (Shanghai, China). They were cultured in RPMI-1640 medium or DMEM media (Gibco, China) supplemented with 10% fetal bovine serum (Gibco, China) at 37 °C in an atmosphere of 5% CO2.

### 4.4 Real-time quantitative reverse transcription PCR

Cells were treated with Trizol (Takara, Japan). Total RNA was extracted using the RNeasy mini kit (Qiagen, United States) and reverse-transcribed with the iScript cDNA Synthesis Kit. iQTM SYBR Green Supermix (Bio-Rad) was performed for qRT-PCR The relative expression of FDX1 was analyzed by the 2-△△Ct method and normalized with GAPDH. FDX1 forward primer: 5ʹ-CTT​TGG​TGC​ATG​TGA​GGG​AA-3ʹ, reverse primer: 5ʹ-GCA​TCA​GCC​ACT​GTT​TCA​GG-3ʹ. GAPDH forward primer: 5ʹ-TGC​ACC​ACC​AAC​TGC​TTA​G-3ʹ, reverse primer 5ʹ-GAT​GCA​GGG​ATG​ATG​TTC-3ʹ.

### 4.5 Protein level analysis

The protein expression level of FDX1 in various cancers was retrieved from the CPTAC Proteomics Database (https://cptac-data-portal.georgetown.edu/). The expression profile data were classified into high and low expression groups according to the median value of FDX1 expression. Then, a total of nine cancer tissues were obtained (HNSC, UCEC, COAD, LUAD, OV, BRCA, PAAD, LIHC and KIRC). Immunohistochemical results of renal carcinoma, seminoma and corresponding normal tissues were obtained from the Human Protein Atlas (Colwill et al., 2011) (HPA: https://www.proteinatlas.org).

### 4.6 Survival analysis

The prognostic value of FDX1 was assessed using Kaplan-Meier analysis between high and low expression groups (Goel et al., 2010). We performed univariate Cox regression analysis to examine the relationship between FDX1 expression and overall survival (OS), disease-related survival (DSS), disease-free interval (DFI), and progression-free interval (PFI) after adjusting for age and tumor stage (Pedersen et al., 2020; Wang et al., 2020). *p* value and hazard ratios (HR) with 95% confidence intervals (CI) were ascertained for each cancer type. Forest plots were generated using the R package “forestplot”.

### 4.7 Assessment of clinical correlations

Clinical correlation between FDX1 and pan-cancer was analyzed containing tumor stage (four stages), age (defined by 60 years old) (Jung et al., 2017), and gender using the R-packages “limma” and “ggpubr”. *p* value <0.05 was considered statistically significant.

### 4.8 Gene set enrichment analysis

Pathways associated with FDX1 were investigated Using “clusterprofiler” package (Yu et al., 2012). In this case, HALLMARK was the referential gene sets. Statistical significance is indicated by an adjusted *p* value <0.05.

### 4.9 Correlation analysis of TMB and MSI with FDX1

Mutation data of FDX1 were all downloaded from the UCSC Xena database and “VarScan2 Variant Aggregation and Masking data” type was selected. Correlation of FDX1 with “Tumor Mutation Burden” (TMB) and “Microsatellite Instability” (MSI) of each cancer was tested using “Spearman” method (Eden et al., 2021), showing in the radar plots. TMB score for each sample was calculated using the TCGA pan-cancer mutation data, while the MSI score was obtained from a previous study (Bonneville et al., 2017).

### 4.10 Tumor microenvironment and immune infiltrate analysis

In order to analyze the infiltration levels of immune cells and stromal cells in pan-cancer, we used ESTIMATE to determine the correlation between FDX1 expression and immune score, stromal score (Yoshihara et al., 2013). Moreover, the relationship between FDX1 expression level and different immune cells (CD8 T cells and monocytes, etc.) was analyzed using the CIBERSORT method (Newman et al., 2015) (http://cibersort.stanford.edu/) and TIMER2 database (Li et al., 2017) (http://timer.cistrome.org/). The p filter was set to less than 0.001.

### 4.11 Co-expression analysis of FDX1 gene

At the same time, the correlation between FDX1 and other genes was evaluated. These genes contained cuproptosis-related genes, immune checkpoint genes, immune-activating genes, immunosuppressive status-related genes, chemokines proteins and chemokine receptors. Heatmaps were used to show the results of the co-expression analyses.

### 4.12 Anticancer drug sensitivity analysis of FDX1 in pan-cancer

RNA-seq data and drug data (Compound activity: DTP NCI-60) were obtained from the CellMiner database (Shankavaram et al., 2009) to evaluate the drug sensitivity of FDX1 in pan-cancer. In order to guarantee the reliability of the results, we only selected anticancer drugs that have been clinically tested or approved by the FDA. Additionally, the ME: MDA_N cell line was excluded from the analysis due to more than 80% of missing values in the drug trial data. The “impute” package was used to impute missing values, and the “ggplot2” and “ggpubr” packages were utilized to draw boxplots.

### 4.13 Docking and molecular dynamics simulations

We used molecular docking simulations to further demonstrate the efficacy of chemotherapeutics and the potential targeting relationship with FDX1. We downloaded the 3D structure of the protein FDX1 (PDB: 3p1m) in the RCSB PDB database (https://www.rcsb.org/), then dehydrated and removed the ligands from the active center by PyMOL software. The small molecule structures of the top three chemotherapeutic drugs Chelerythrine, INK-128 and Defactinib (Everolimus has no structures available) were downloaded from the zinc15 database (https://zinc.docking.org/).AutoDockTools 1.5.6 was used to work with receptor proteins and small molecule ligands, such as adding polar hydrogens, charge calculations and setting up rotation bonds. The parameters of the receptor protein docking site were set to include the active pocket site for small molecule ligand binding. Grid box centred at (28.296, 42.2, 26.794) Å, the grid lengths in XYZ directions were 104, 124 and 104 Å, respectively. Finally, the receptor protein was docked with the small molecule ligand by using Autodock4, and the docking results were displayed by PyMOL software.

### 4.14 Statistical analysis

All statistical analyses were performed by the R (https://www.r-project.org/). *p* < 0.05 was considered statistically significant, and we have marked * in the results of the different analyses, where * represents *p* < 0.05, ** represent *p* < 0.01, and *** represent *p* < 0.001.

## Data Availability

The original contributions presented in the study are included in the article/Supplementary Materials, further inquiries can be directed to the corresponding author.

## Nomenclature

### Abbreviations

ACC: Adrenocortical carcinoma
KIRC: Kidney renal clear cell carcinoma
HNSC: Head and Neck squamous cell carcinoma
THCA: Thyroid carcinoma
LGG: Brain Lower Grade Glioma
TMB: Tumor Mutation Burden
MSI: Microsatellite Instability
FDXR: ferredoxin reductase
DEG: differentially expressed gene
OS: overall survival
DSS: disease-related survival
DFI: disease-free interval
PFI: progression-free interval
HR: hazard ratios
CI: confidence intervals
BRCA: Breast invasive carcinoma
CHOL: Cholangiocarcinoma
COAD: Colon adenocarcinoma
KICH Kidney Chromophobe
KIRP: Kidney renal papillary cell carcinoma
LIHC: Liver hepatocellular carcinoma
LUAD: Lung adenocarcinoma
LUSC: Lung squamous cell carcinoma
PCPG: Pheohromocytoma and Paraganglioma
READ: Rectum adenocarcinoma
SARC: Sarcoma
PAAD: Pancreatic adenocarcinoma
UCEC: Uterine Corpus Endometrial Carcinoma
OV: Ovarian serous cystadenocarcinoma
CESC: Cervial squamous cell carcinoma and endocervical adenocarcinoma
SKCM: Skin Cutaneous Melanoma
MESO Mesothelioma
ESCA: Esophageal carcinoma
TGCT: Testicular Germ Cell Tumors
THYM: Thymoma
PRAD: Prostate adenocarcinoma
BLCA: Bladder Urothelial Carcinoma
STAD: Stomach adenocarcinoma
DLBC: Lymphoid Neoplasm Diffuse Large B-cell Lymphoma
UVM: Uveal Melanoma
