# Supplementary material for: Pan-cancer analyses confirmed the cuproptosis-related gene FDX1 as an immunotherapy predictor and prognostic biomarker
Source: Front Genet. 2022 Aug 5;13:923737. doi: 10.3389/fgene.2022.923737 (PMC9388757; doi:10.3389/fgene.2022.923737)
Supplement: Supplementary file 1 [file DataSheet1.ZIP › Supplementary_Material/Supplementary_Material.docx]

Supplementary Material

# Supplementary Figure Legends

**Supplementary Figure 1.** Complementary differential expression of FDX1 in pan-cancer without paracancerous tissue by the GEPIA platform. The red boxplot represents tumor (T) and the blue boxplot represents normal tissue (N).

**Supplementary Figure 2.** K-M analysis of the association between FDX1 levels and OS (A-D), DSS (E), DFI (F) and PFI (G-H).

**Supplementary Figure 3.** Results of GSEA analysis associated with FDX1 expression by the HALLMARK gene set: (A)ESCA, (B)KIRC, (C)KIRP, (D)LIHC, (E)LUSC, (F)PRAD, (G)THYM.

**Supplementary Figure 4.** Results of GSEA analysis associated with FDX1 expression by the HALLMARK gene set: (A)BLCA, (B)COAD, (C)LUAD, (D)READ.

**Supplementary Figure 5.** The correlation between FDX1 expression and immune cell infiltration.

**Supplementary Figure 6.** Gene co-expression analysis of FDX1 in pan-cancer. The heatmaps presenting the correlations of FDX1 expression with genes related to celluar redox signaling.

# Supplementary Tables

**Table S1.** Detailed results of correlation analysis of FDX1 expression with TMB in a pan-cancer cohort.

| CancerType | cor | pValue | sig |
| --- | --- | --- | --- |
| ACC | 0.179461745 | 0.113525478 |  |
| BLCA | 0.064093423 | 0.196363924 |  |
| BRCA | -0.044114551 | 0.16892332 |  |
| CESC | 0.045235399 | 0.446036184 |  |
| CHOL | -0.108463267 | 0.528907624 |  |
| COAD | -0.016086003 | 0.74963844 |  |
| DLBC | 0.107396871 | 0.525554631 |  |
| ESCA | 0.230063293 | 0.003426442 | ** |
| GBM | -0.069641529 | 0.400312108 |  |
| HNSC | 0.106098735 | 0.018570337 | * |
| KICH | -0.249652854 | 0.044899874 | * |
| KIRC | -0.140355309 | 0.010454083 | * |
| KIRP | -0.01966726 | 0.744066652 |  |
| LAML | -0.086165541 | 0.501919364 |  |
| LGG | 0.230624793 | 1.79E-07 | *** |
| LIHC | -0.054090309 | 0.30676233 |  |
| LUAD | -0.155234925 | 0.000475666 | *** |
| LUSC | 0.086359379 | 0.056596839 |  |
| MESO | -0.16077246 | 0.156943603 |  |
| OV | 0.068695292 | 0.258868966 |  |
| PAAD | 0.055542096 | 0.498174862 |  |
| PCPG | -0.122325483 | 0.104803073 |  |
| PRAD | 0.16459326 | 0.000284566 | *** |
| READ | 0.154971124 | 0.0760163 |  |
| SARC | 0.058591415 | 0.371231747 |  |
| SKCM | 0.006656021 | 0.886175844 |  |
| STAD | 0.224246107 | 1.41E-05 | *** |
| TGCT | 0.02668334 | 0.750040667 |  |
| THCA | -0.172464244 | 0.000141756 | *** |
| THYM | -0.400100743 | 7.84E-06 | *** |
| UCEC | 0.18793757 | 1.46E-05 | *** |
| UCS | 0.206322366 | 0.127104826 |  |
| UVM | -0.170230065 | 0.131129441 |  |

**Table S2.** Detailed results of correlation analysis of FDX1 expression with MSI in a pan-cancer cohort.

| CancerType | cor | pValue | sig |
| --- | --- | --- | --- |
| ACC | -0.263136114 | 0.01913047 | * |
| BLCA | -0.057748879 | 0.244479582 |  |
| BRCA | 0.005806896 | 0.852264321 |  |
| CESC | -0.092479518 | 0.108736796 |  |
| CHOL | -0.038095238 | 0.825089749 |  |
| COAD | -0.057215424 | 0.238080085 |  |
| DLBC | 0.526855749 | 0.000119576 | *** |
| ESCA | 0.110028281 | 0.166035187 |  |
| GBM | -0.123397039 | 0.131170316 |  |
| HNSC | 0.148821394 | 0.000885522 | *** |
| KICH | -0.025942237 | 0.837471156 |  |
| KIRC | 0.230224233 | 2.09E-05 | *** |
| KIRP | 0.06262903 | 0.292025885 |  |
| LAML | -0.106826228 | 0.385906661 |  |
| LGG | 0.028891167 | 0.515882568 |  |
| LIHC | -0.07500674 | 0.150442503 |  |
| LUAD | -0.132169628 | 0.00275742 | ** |
| LUSC | -0.193868765 | 1.46E-05 | *** |
| MESO | 0.106364221 | 0.341561736 |  |
| OV | -0.105957595 | 0.081095769 |  |
| PAAD | -0.251048077 | 0.000804956 | *** |
| PCPG | -0.104665619 | 0.16440825 |  |
| PRAD | 0.006371169 | 0.88755978 |  |
| READ | 0.031713406 | 0.6981203 |  |
| SARC | -0.026669344 | 0.672896298 |  |
| SKCM | -0.05624595 | 0.224559296 |  |
| STAD | 0.243779204 | 1.83E-06 | *** |
| TGCT | -0.119594601 | 0.144919724 |  |
| THCA | -0.038429726 | 0.395497834 |  |
| THYM | -0.117453918 | 0.205268473 |  |
| UCEC | 0.185674503 | 1.46E-05 | *** |
| UCS | -0.115554189 | 0.396398415 |  |
| UVM | 0.025881319 | 0.81973611 |  |

**Table S3.** Drug sensitivity analysis of FDX1

| Drug | cor | pvalue | Mechanism of action c |
| --- | --- | --- | --- |
| 6-Bromoindirubin-3'-Acetoxime | -0.266570067 | 0.041266346 | PK:GSK3A |
| AMONAFIDE | 0.266252042 | 0.041518223 | TOP2 |
| Avagacestat | -0.256576817 | 0.049807838 | PSM\|APH1\|Gamma secretase |
| AZD-3147 | -0.317546088 | 0.014256898 | PK:MTOR |
| AZD-6738 | -0.289516853 | 0.026141117 | PK:ATR |
| AZD-6738 ( R,S-isomer) | -0.259177639 | 0.047456913 | PK:ATR |
| AZD-8055 | -0.275188234 | 0.034905771 | PK:STK,MTOR |
| AZD-8186 | -0.265794556 | 0.041882777 | PI3KB\|PK:PIK3 |
| BPTES | -0.291716914 | 0.02497544 | - |
| CB-839 | -0.256867518 | 0.049540441 | GLS |
| CC-115 | -0.295193814 | 0.023222684 | PK:MTOR |
| CC-223 | -0.285897365 | 0.028158347 | PK:MTOR |
| Chelerythrine | 0.366634274 | 0.004288778 | BCL2\|PK:PRKCA,STK |
| Defactinib | -0.350089761 | 0.006564685 | PK:FAK |
| enantiomer of PF-4176340 | -0.28361159 | 0.029498325 | PK:AKT |
| ENMD-2076 Precursor | -0.269108775 | 0.039300506 | PK:STK,AURK |
| Everolimus | -0.351616456 | 0.006317571 | PK:STK,MTOR |
| futibutinib | -0.281350449 | 0.030875977 | PK:FGFR |
| GDC-0349 | -0.313641232 | 0.015565008 | PK:MTOR |
| GSK-2141795 | -0.260667406 | 0.046151716 | PK:AKT |
| GSK-2606414 | -0.304321597 | 0.019108641 | PK:EIF2AK3 |
| Ifosfamide | 0.346768606 | 0.007131654 | A7\|AlkAg |
| INCB-047775 | -0.28282995 | 0.029968618 | PK:AKT |
| INK-128 | -0.352613774 | 0.006160578 | PK:MTOR |
| JNJ-42756493 | -0.309055409 | 0.017231237 | PK:FGFR,FGFR1,FGFR2,FGFR3,FGFR4 |
| KPT-9274 | 0.302449434 | 0.01989774 | PK:PAK4 |
| KU-55933 | -0.318274266 | 0.014023705 | PK:ATM |
| LY-3023414 | -0.348786933 | 0.006782212 | PK:PIK3,MTOR |
| M2698 | -0.30872102 | 0.017358477 | PK:AKT |
| MK-2206 | -0.265622419 | 0.042020627 | PK:STK,AKT,AKT1,AKT2,AKT3 |
| Nelarabine | 0.275251485 | 0.03486229 | Ds |
| Pp-242 | -0.268484168 | 0.03977684 | PK:MTOR |
| PQR-309 | -0.272691925 | 0.036658172 | PK:PIK3,MTOR |
| PQR-620 | -0.339924655 | 0.008436378 | PK:MTOR |
| PX-316 | 0.290862358 | 0.025422904 | PK:STK |
| PYRAZOLOACRIDINE | 0.282216634 | 0.030342012 | Db |
| RG-7741 | -0.289325816 | 0.02624446 | - |
| Ribavirin | 0.306385958 | 0.018269554 | - |
| tic10 | 0.259883151 | 0.046835092 | - |
| UMI-77 | 0.259903459 | 0.046817293 | MCL1 |
| VE-821 | -0.293305378 | 0.02416131 | PK:STK,ATR |
| Vorinostat | 0.273293193 | 0.036229553 | HDAC |
| ZSTK-474 | -0.266148392 | 0.041600588 | PK:PIK3 |
