# Supplementary figures and images for: Pan-cancer analyses confirmed the cuproptosis-related gene FDX1 as an immunotherapy predictor and prognostic biomarker
Source: Front Genet. 2022 Aug 5;13:923737. doi: 10.3389/fgene.2022.923737 (PMC9388757; doi:10.3389/fgene.2022.923737)

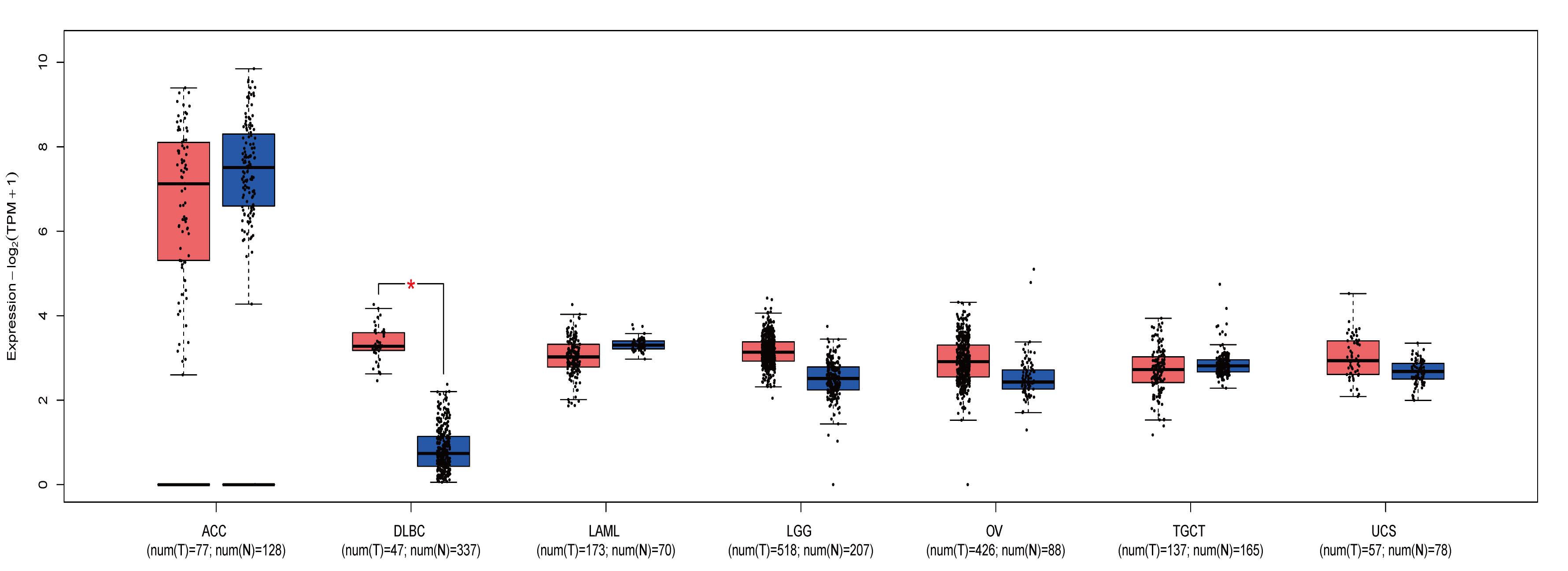

Supplement: Supplementary file 1 [file DataSheet1.ZIP › Supplementary_Material/Figure S1.tif]

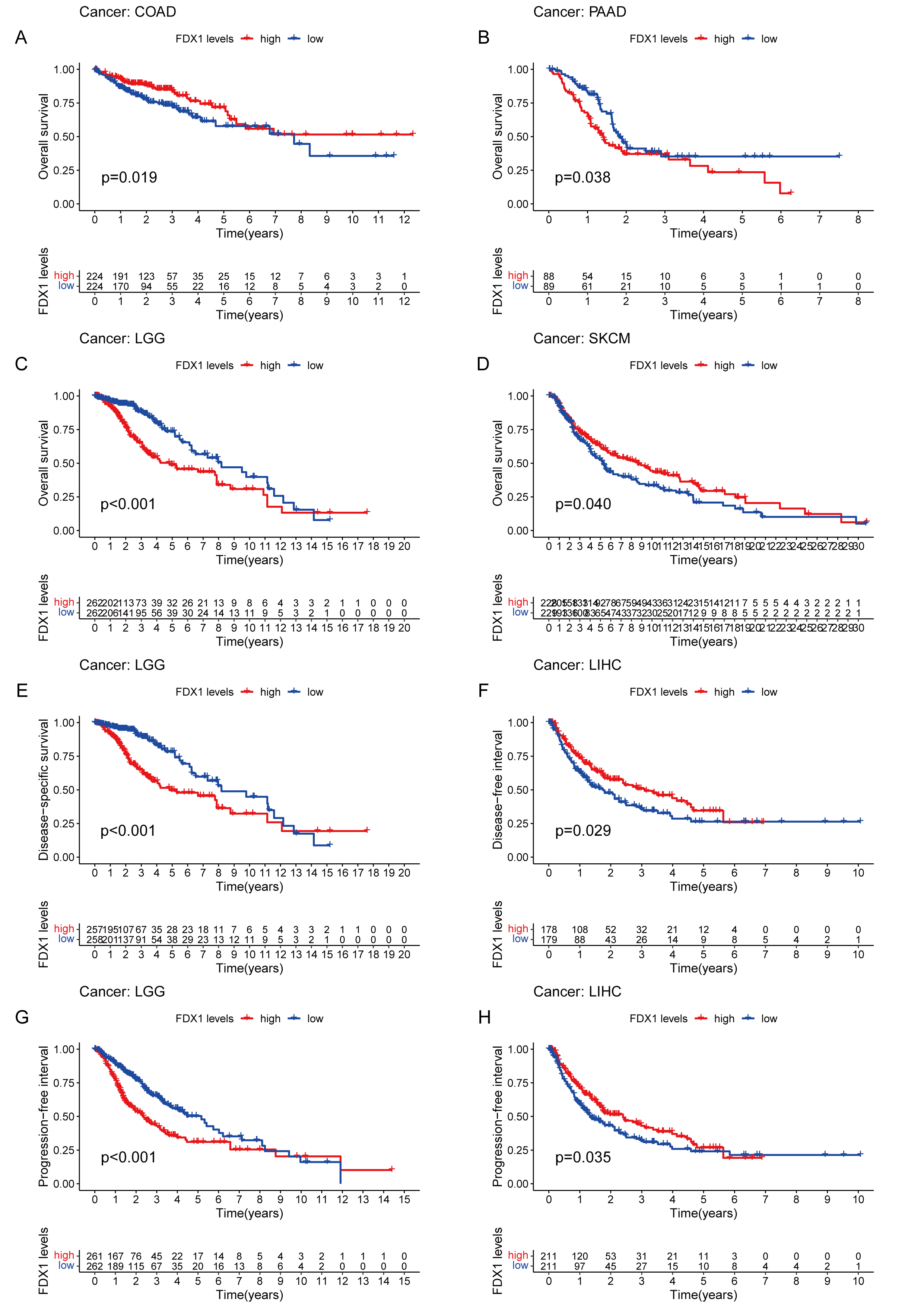

Supplement: Supplementary file 1 [file DataSheet1.ZIP › Supplementary_Material/Figure S2.tif]

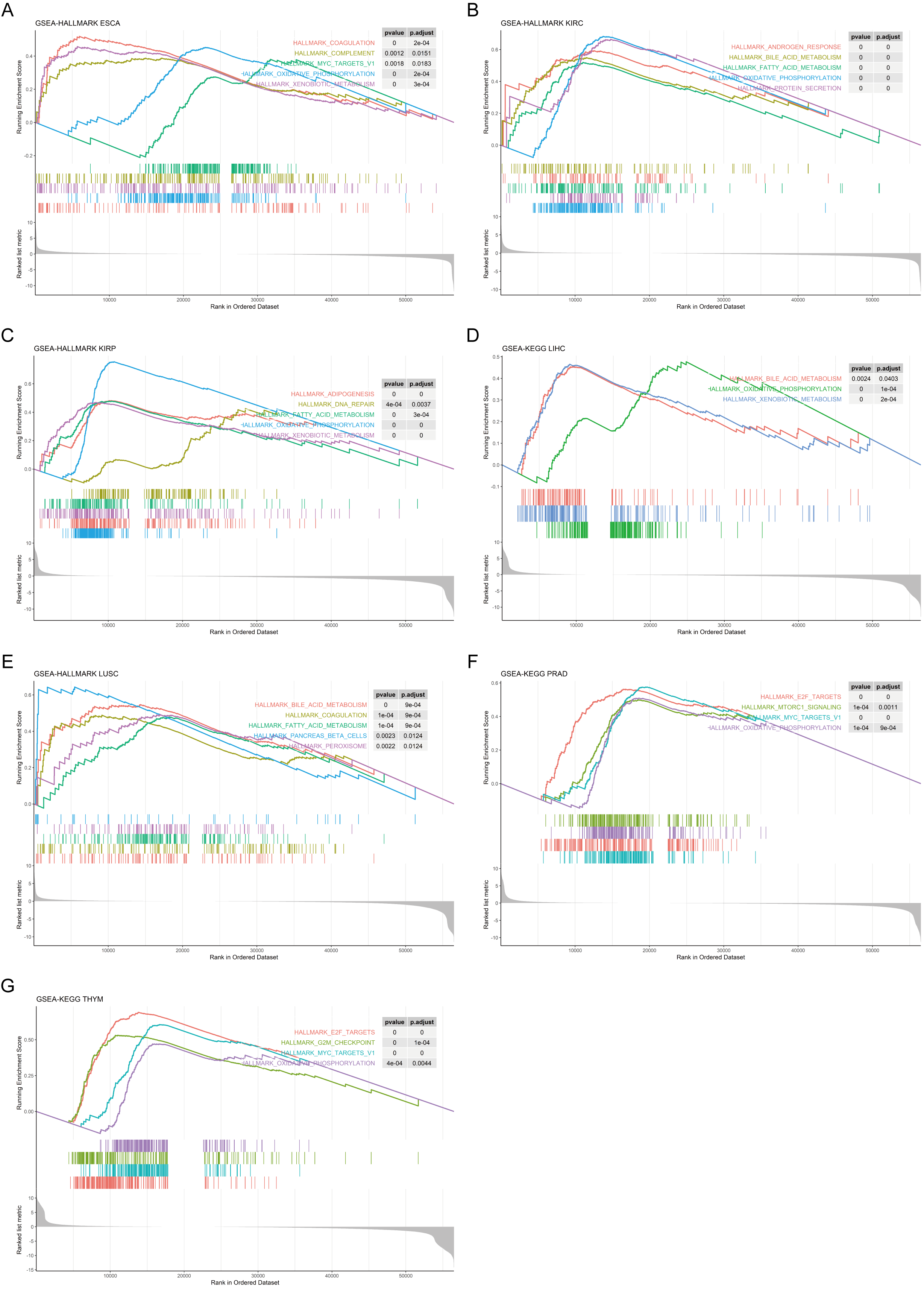

Supplement: Supplementary file 1 [file DataSheet1.ZIP › Supplementary_Material/Figure S3.tif]

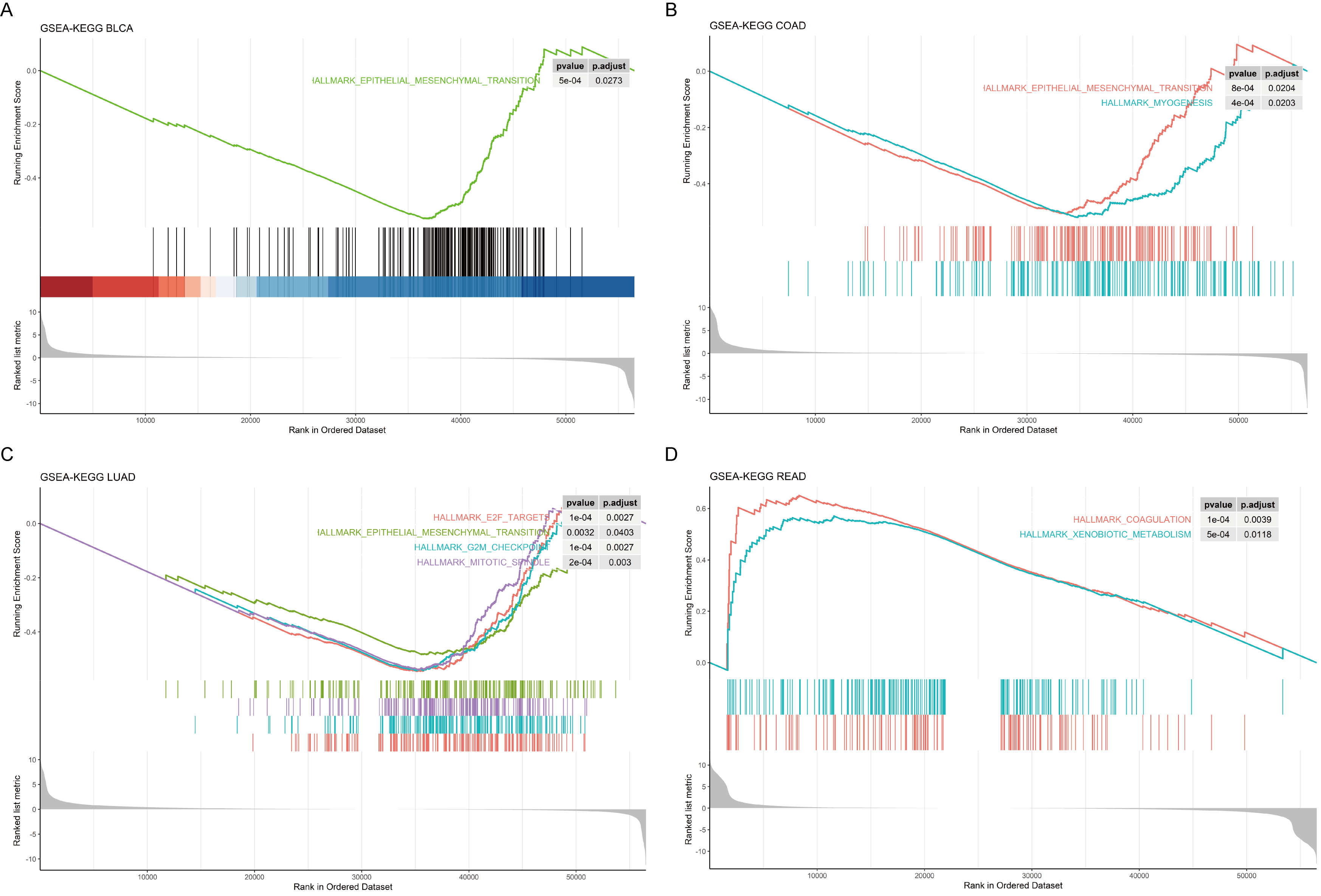

Supplement: Supplementary file 1 [file DataSheet1.ZIP › Supplementary_Material/Figure S4.tif]

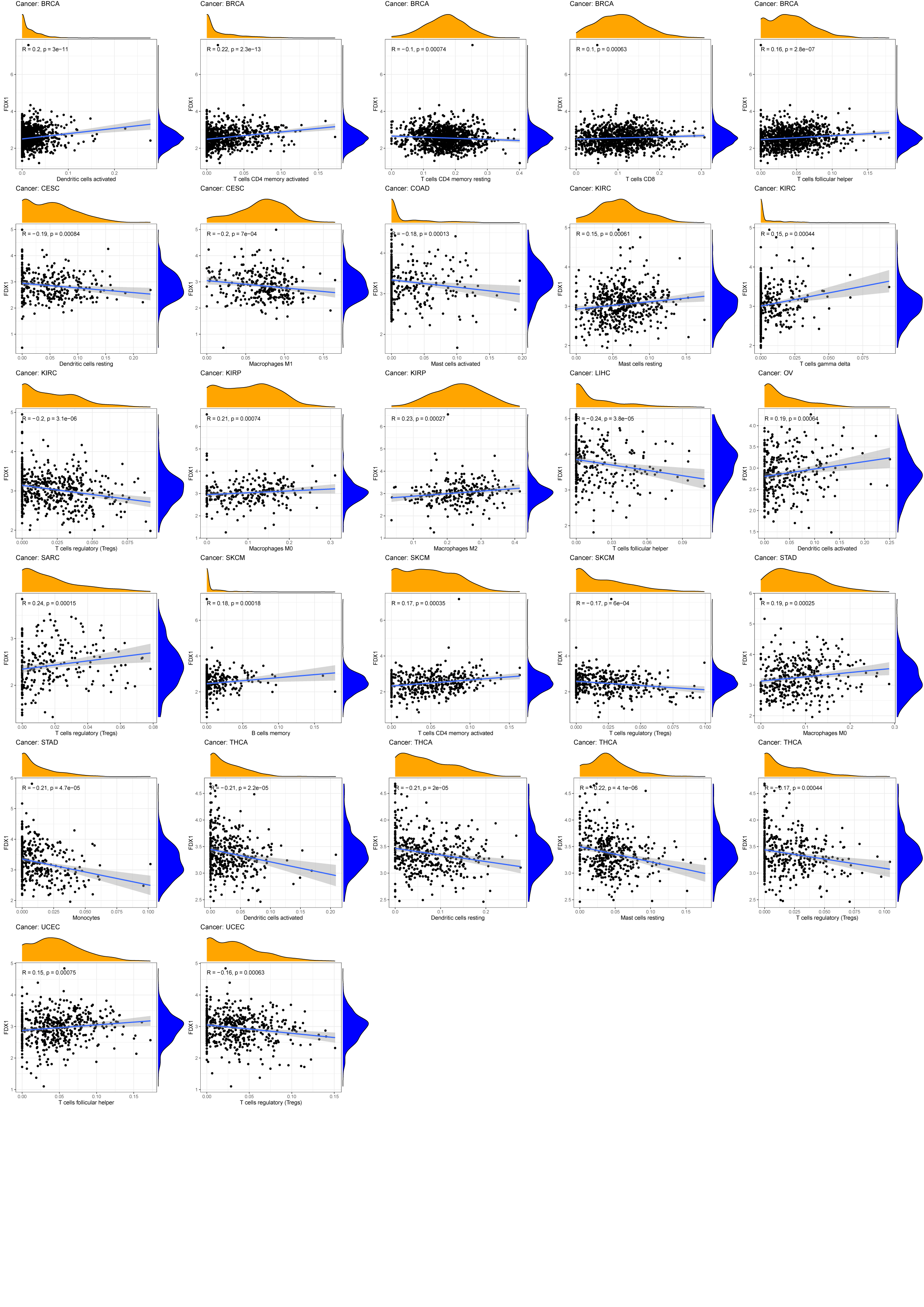

Supplement: Supplementary file 1 [file DataSheet1.ZIP › Supplementary_Material/Figure S5.tif]

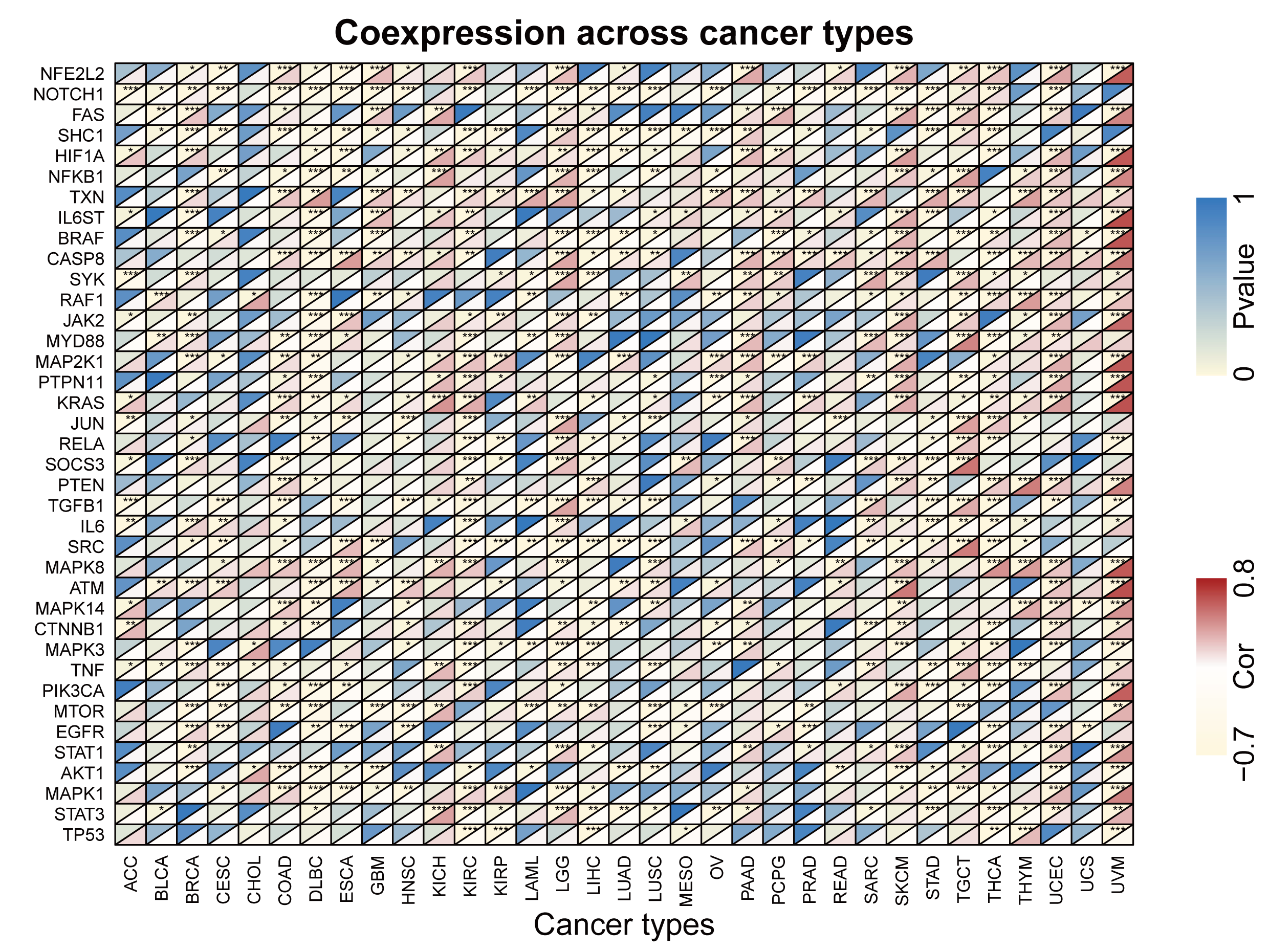

Supplement: Supplementary file 1 [file DataSheet1.ZIP › Supplementary_Material/Figure S6.tif]

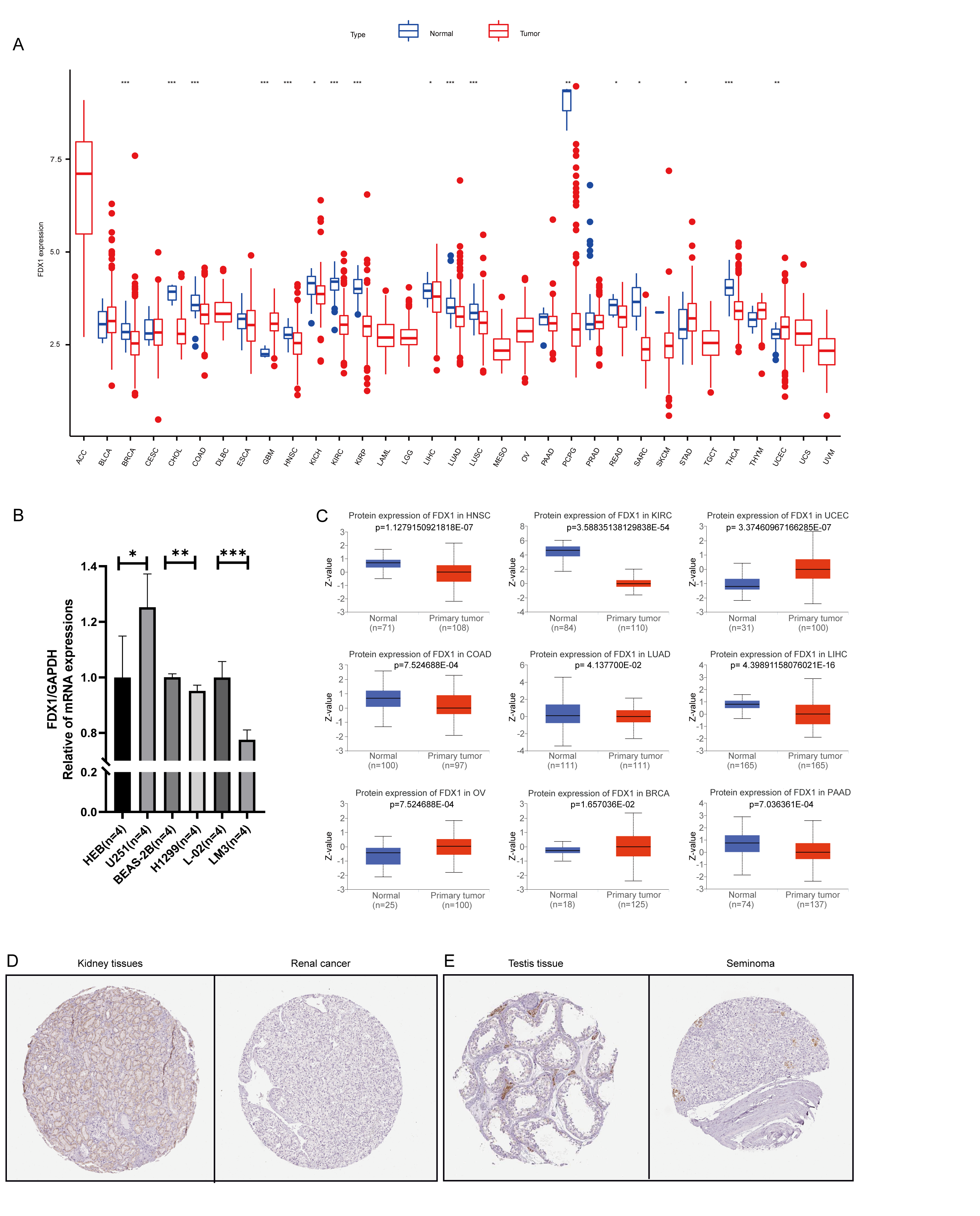

Supplement: Supplementary file 1 [file DataSheet1.ZIP › Supplementary_Material/Figure1.tif]

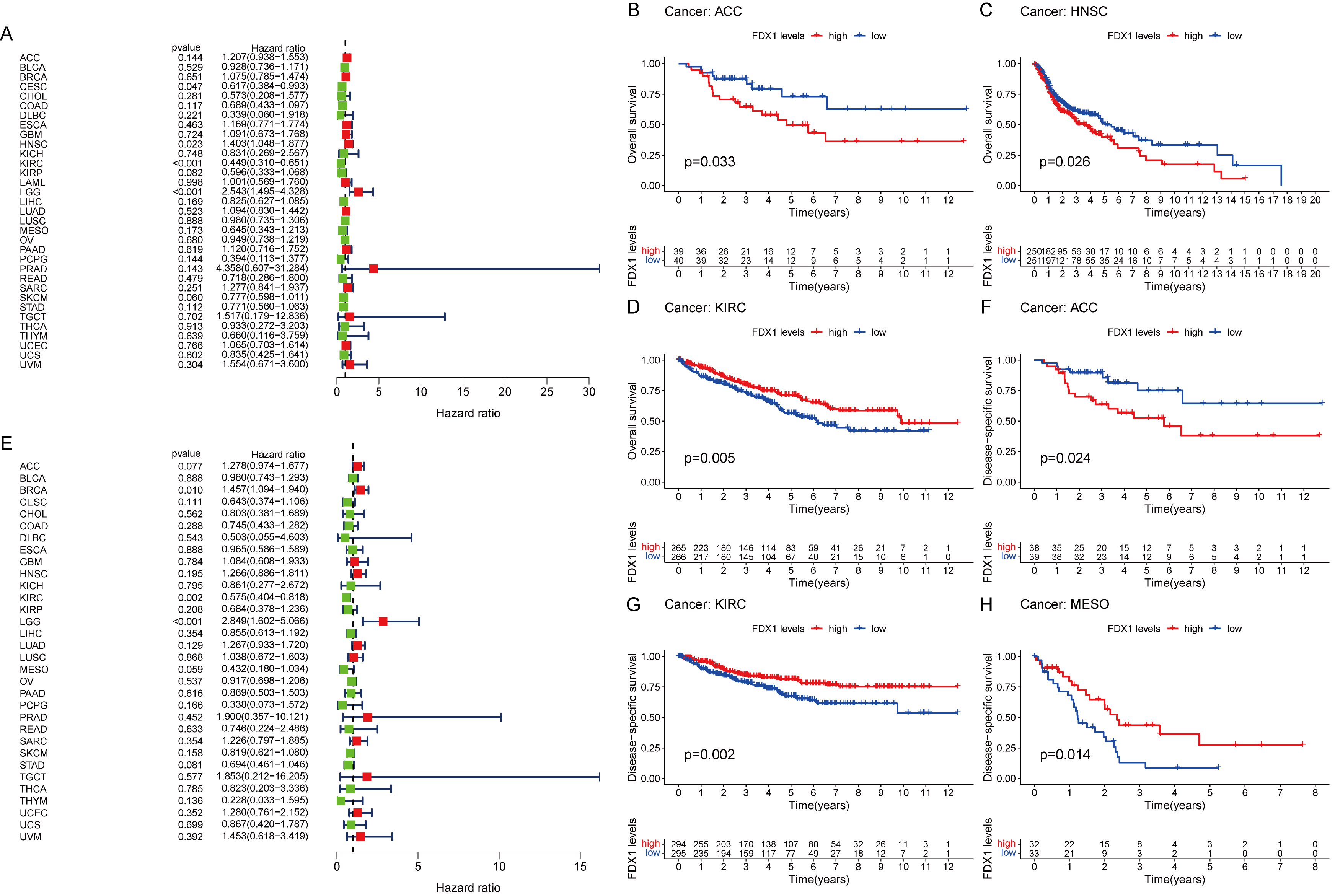

Supplement: Supplementary file 1 [file DataSheet1.ZIP › Supplementary_Material/Figure2.tif]

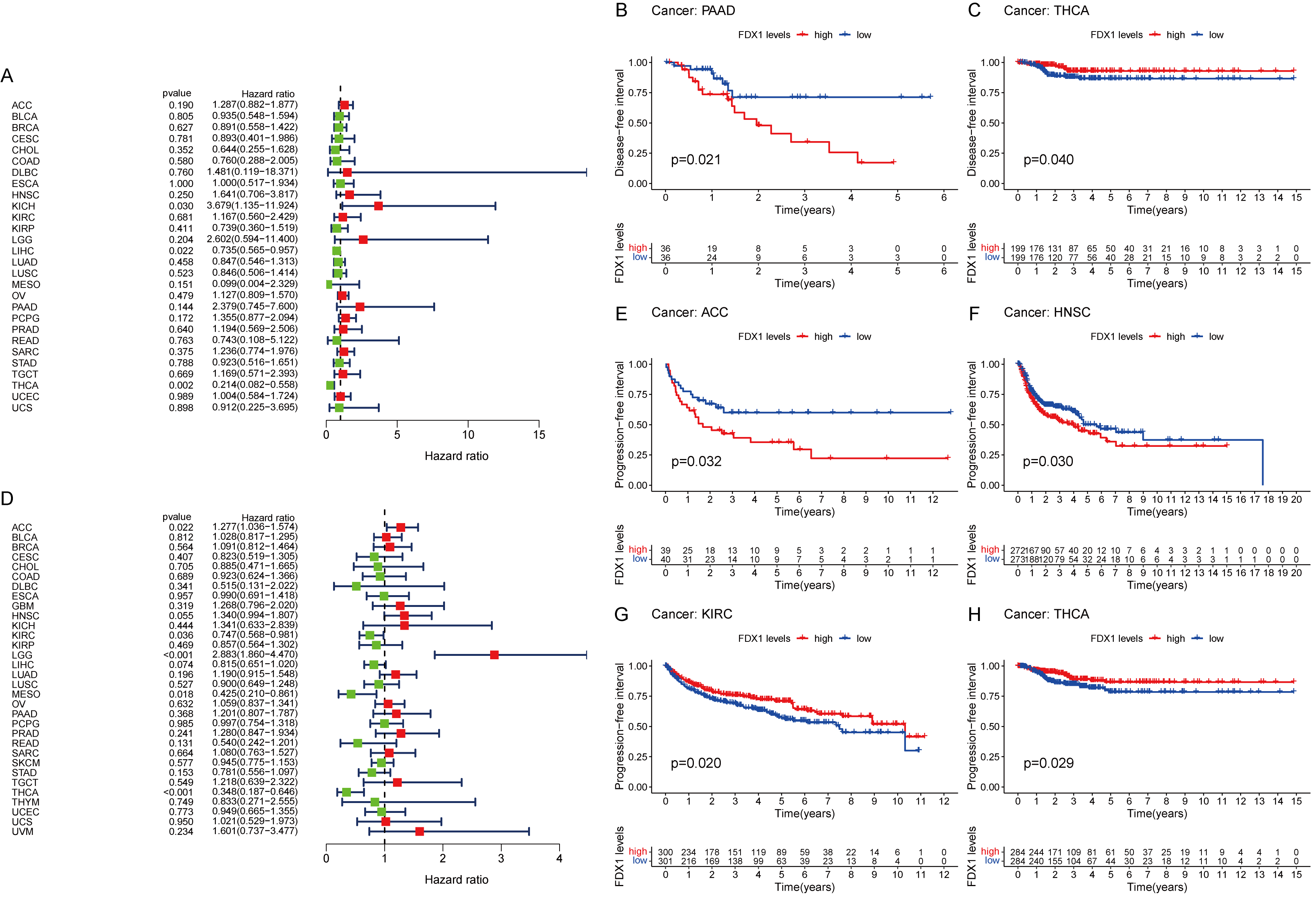

Supplement: Supplementary file 1 [file DataSheet1.ZIP › Supplementary_Material/Figure3.tif]

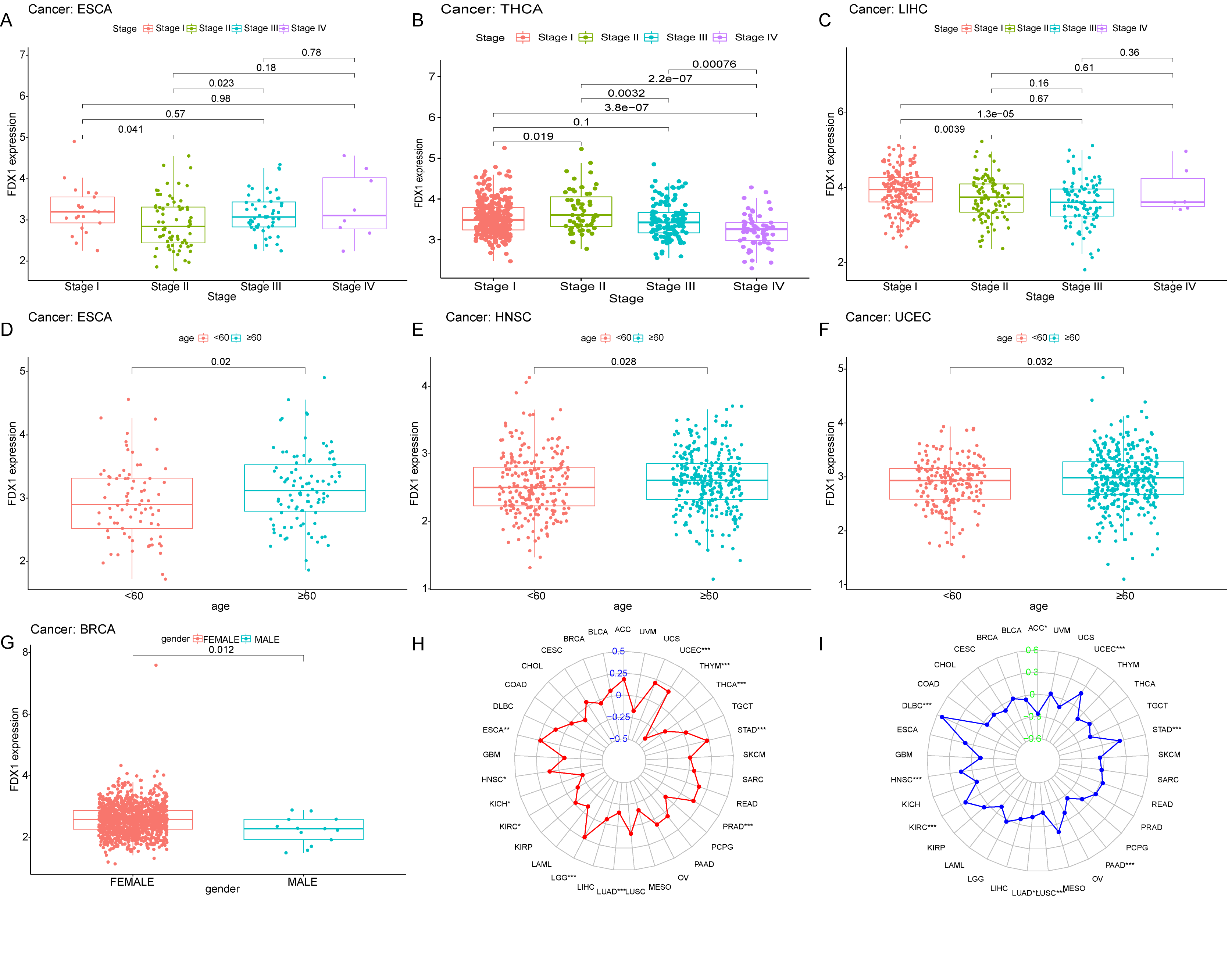

Supplement: Supplementary file 1 [file DataSheet1.ZIP › Supplementary_Material/Figure4.tif]

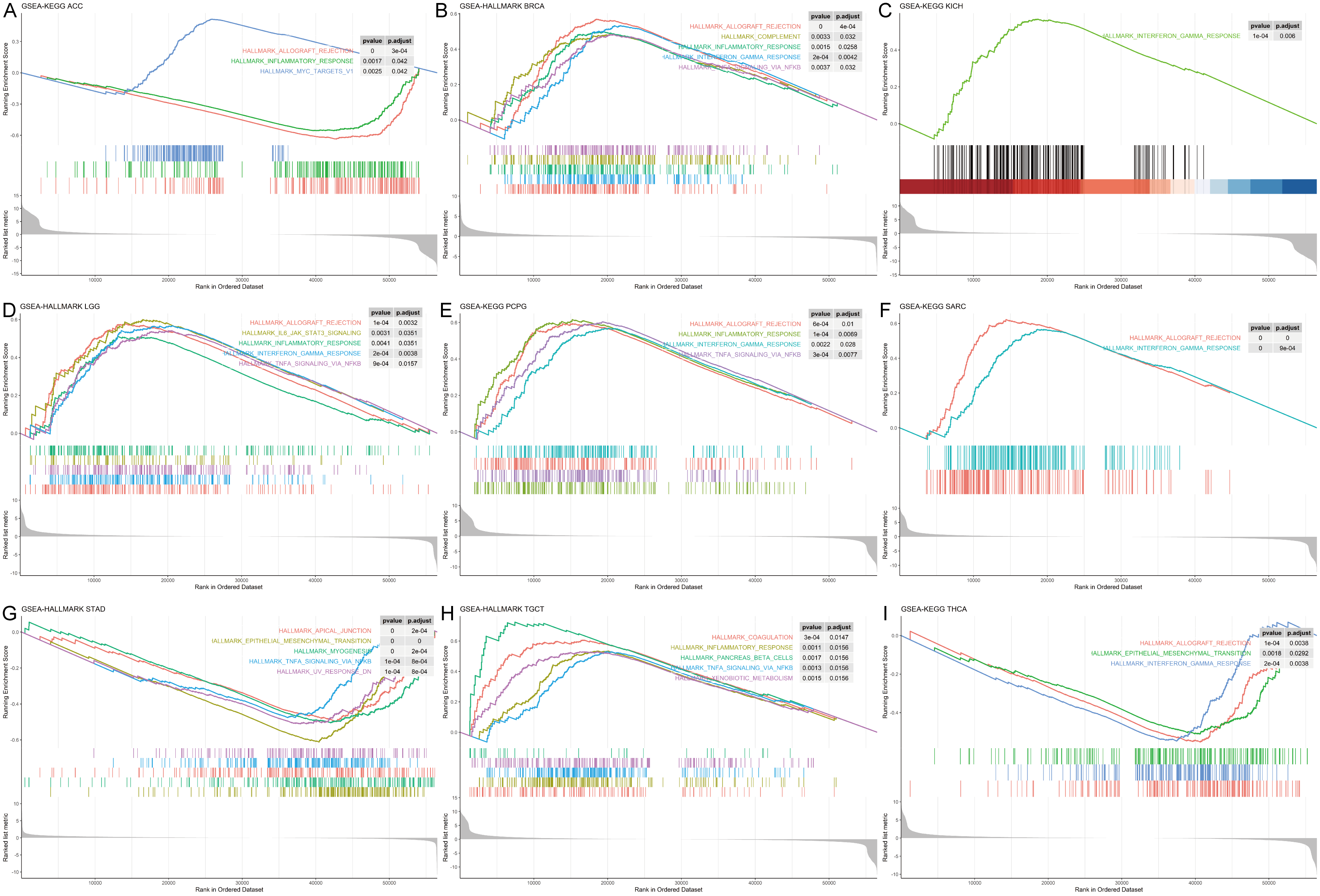

Supplement: Supplementary file 1 [file DataSheet1.ZIP › Supplementary_Material/Figure5 .tif]

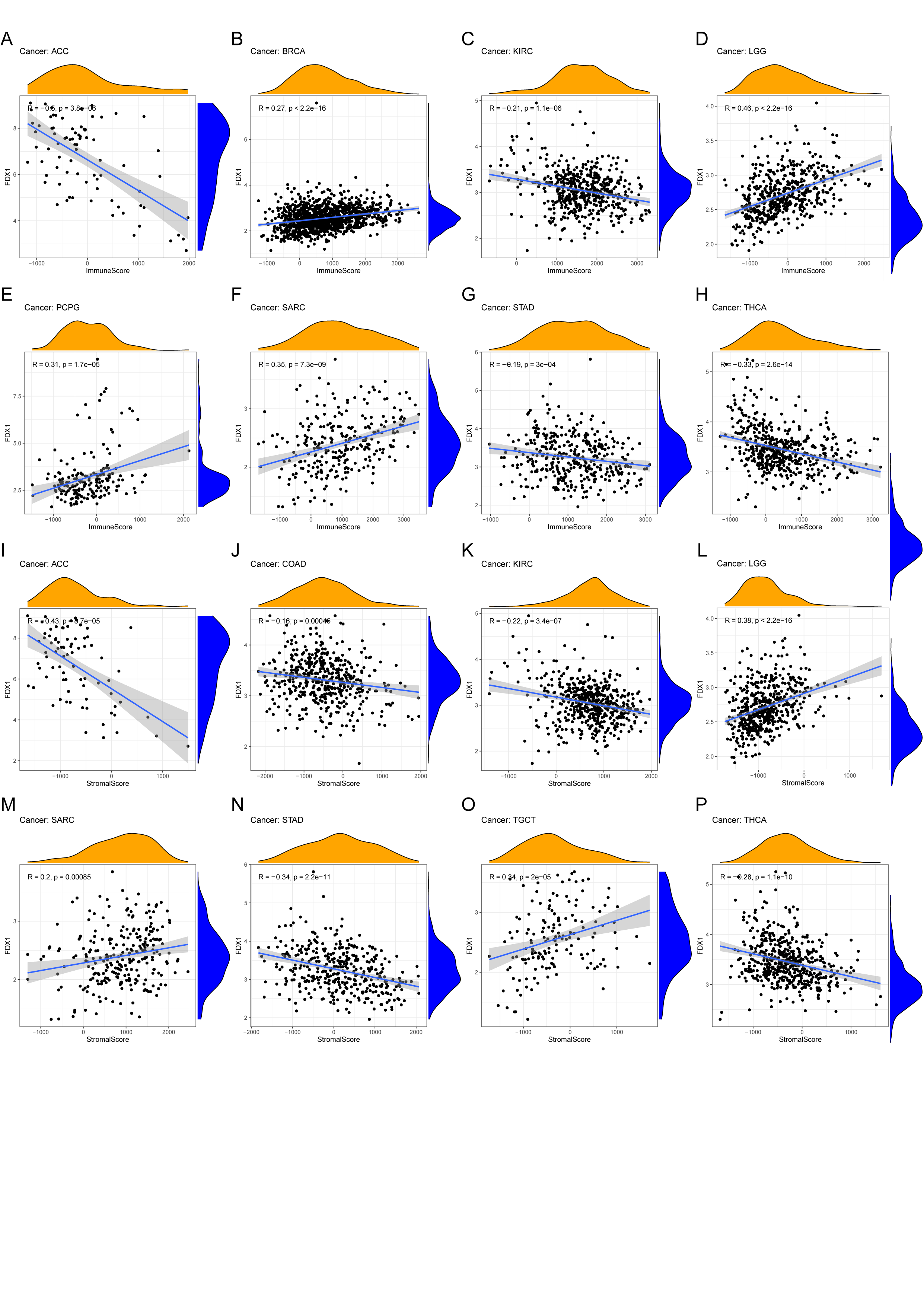

Supplement: Supplementary file 1 [file DataSheet1.ZIP › Supplementary_Material/Figure6.tif]

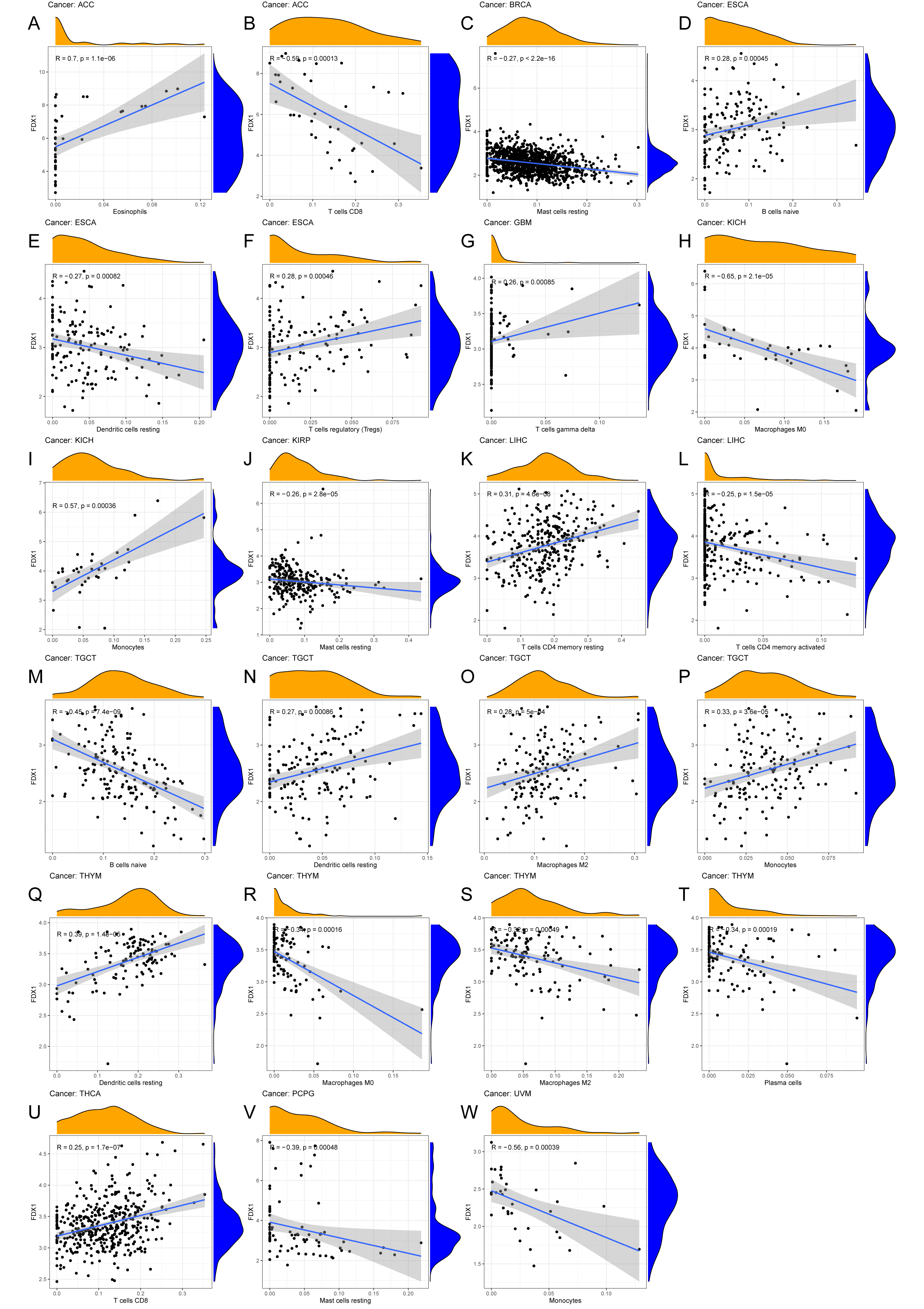

Supplement: Supplementary file 1 [file DataSheet1.ZIP › Supplementary_Material/Figure7.tif]

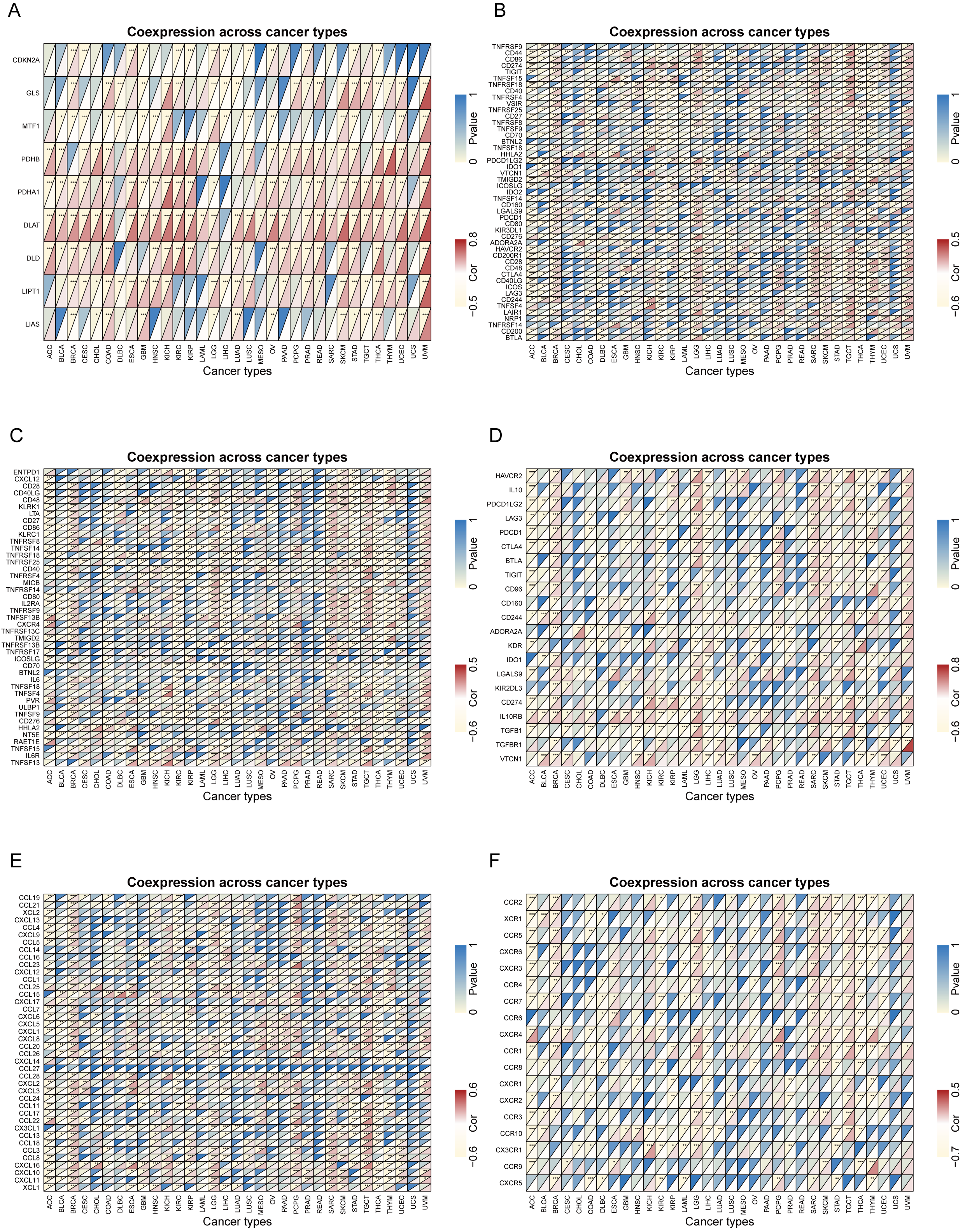

Supplement: Supplementary file 1 [file DataSheet1.ZIP › Supplementary_Material/Figure8.tif]

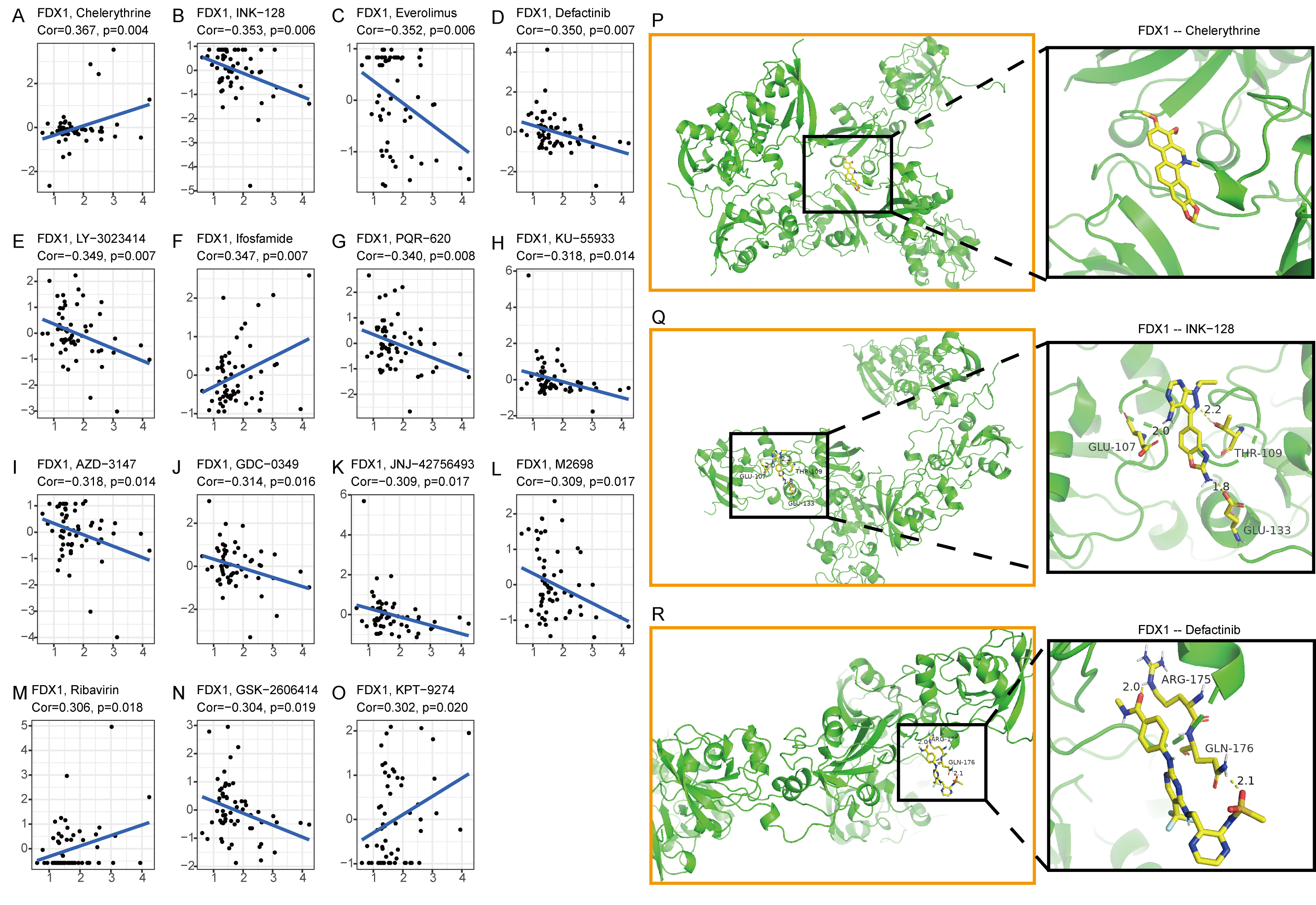

Supplement: Supplementary file 1 [file DataSheet1.ZIP › Supplementary_Material/Figure9 .tif]
